# Alu retrotransposons modulate Nanog expression through dynamic changes in regional chromatin conformation via aryl hydrocarbon receptor

**DOI:** 10.1186/s13072-020-00336-w

**Published:** 2020-03-14

**Authors:** Francisco J. González-Rico, Cristina Vicente-García, Almudena Fernández, Diego Muñoz-Santos, Lluís Montoliu, Antonio Morales-Hernández, Jaime M. Merino, Angel-Carlos Román, Pedro M. Fernández-Salguero

**Affiliations:** 1 Departamento de Bioquímica y Biología Molecular, Facultad de Ciencias, Universidad de Extremadura, Av. de Elvas s/n, 06071 Badajoz, Spain; 2 Department of Molecular and Cellular Biology, Centro Nacional de Biotecnología (CNB), Consejo Superior de Investigaciones Científicas (CSIC), Campus de Cantoblanco, C/Darwin 3, 28049 Madrid, Spain; 3 Centro de Investigación Biomédica en Red de Enfermedades Raras (CIBERER), ISCIII, Madrid, Spain

**Keywords:** Alu retrotransposons, Aryl hydrocarbon receptor, Differentiation, Nanog, Chromatin conformation

## Abstract

Transcriptional repression of Nanog is an important hallmark of stem cell differentiation. Chromatin modifications have been linked to the epigenetic profile of the Nanog gene, but whether chromatin organization actually plays a causal role in Nanog regulation is still unclear. Here, we report that the formation of a chromatin loop in the Nanog locus is concomitant to its transcriptional downregulation during human NTERA-2 cell differentiation. We found that two Alu elements flanking the Nanog gene were bound by the aryl hydrocarbon receptor (AhR) and the insulator protein CTCF during cell differentiation. Such binding altered the profile of repressive histone modifications near Nanog likely leading to gene insulation through the formation of a chromatin loop between the two Alu elements. Using a dCAS9-guided proteomic screening, we found that interaction of the histone methyltransferase PRMT1 and the chromatin assembly factor CHAF1B with the Alu elements flanking Nanog was required for chromatin loop formation and Nanog repression. Therefore, our results uncover a chromatin-driven, retrotransposon-regulated mechanism for the control of Nanog expression during cell differentiation.

## Introduction

Cellular differentiation is a key process during embryonic development [1, 2] and in adult stem cell homeostasis [3] whose alteration can lead to pathological states including cancer [4, 5]. In the last few years, transcriptional regulatory mechanisms that control pluripotency and differentiation have been described (see review in [6]). Taking advantage of this knowledge, specific techniques have been recently developed to revert differentiated cells to an induced pluripotent stem cell phenotype [7, 8]. Some of these transcription factors are considered master regulators of pluripotency and include Nanog, Oct4, Sox2, c-Myc and KLF4, among others [6]. Nanog was first described as an embryo-specific homeobox gene [9]. Later on, two independent groups found that it was required for the maintenance of pluripotency in embryonic stem cells [10, 11], in which it acts as transcriptional activator of genes related to pluripotency and as transcriptional repressor of genes involved in differentiation [12]. Nanog expression can be self-induced [12] in embryonic stem cells or activated by different transcription factors like Oct4 and Sox2 [13] or FoxD3 [14]. Upon differentiation, proteins such as p53, Foxa1, RIP140 or the aryl hydrocarbon receptor (AhR) are also able to repress Nanog [15–18]. These changes in Nanog expression and in the levels of other pluripotency regulators involve epigenetic modifications of their loci, as observed in several differentiation models [18–23]. Under stem cell differentiating conditions, chromatin folding is altered, the chromatin of topologically associated domains (TADs) is reordered and the whole transcriptome of the cell rewired [24–28]. Nevertheless, we are still far from a comprehensive view of the molecular events that comprise cellular differentiation and on the functions of specific regulators of pluripotency. We recently found that the transcription factor AhR was required for retinoic acid (RA)-mediated differentiation of NTERA-2 cells (hereafter NTERA) [17]. Specifically, RA-induced differentiation promoted AhR binding to Alu retrotransposons flanking pluripotency genes Nanog and Oct4. Notably, Alu-generated transcripts in differentiated cells were able to repress Nanog and Oct4 expression by a mechanism involving the miRNA machinery [17]. In addition, these results are in agreement with the proposed roles for repetitive elements as enhancer–promoter insulators and/or chromatin barriers and architecture [29–34]. In this work, we have investigated if Alu retrotransposons located near pluripotency genes could participate in human stem cell differentiation by modulating chromatin structure and dynamics. To test such possibility, we focused on the changes in chromatin folding surrounding the Nanog locus that could take place during NTERA differentiation. Our main conclusion is that a molecular complex, composed by AhR, PRMT1, CHAF1B and CTCF, interacts with Alu elements modifying the epigenetic profile and generating a chromatin loop around the Nanog gene that will lead to its repression during RA-mediated differentiation. In fact, impairing the interaction between AhR and PRMT1 with the Alu elements restored Nanog expression in differentiation-induced cells.

## Results

### Alu elements located flanking the Nanog locus have enhancer-blocking activity

We have recently found that transcriptional downregulation of Nanog during NTERA cell differentiation was dependent on the upregulation of AhR and on its binding to repetitive sequences neighboring the Nanog locus, being those 7SL RNA-derived human retroelements (Alu family) [17]. Following the same bioinformatic algorithm used to analyze the mouse heterologous of these human repetitive elements (e.g., B1 family) [35], we have extracted those Alu retrotransposons located in human gene promoters that have an AhR binding site (xenobiotic response element, XRE) and an E-Box binding site separated by a conserved nucleotide sequence (Additional file 1: Figure S1A). We have identified three major classes of elements in which an XRE motif is separated from an E-box by exactly 14, 36 or 45 bp (so-called X14S, X36S and X45S Alus) (Additional file 1: Figure S1A). Notably, further gene analysis revealed that these Alu elements were highly represented in the 3′ and 5′ flanking regions of stemness-relevant human genes including Nanog, Oct4, Sox2, Notch1 and KLF4, among others (Additional file 1: Figure S1B). For Nanog, X45S and X14S Alus were present in its 5′ and 3′ flanking regions, respectively (Additional file 1: Figure S1B). Therefore, we decided to investigate the molecular mechanisms by which these retrotransposons could repress stemness-relevant genes during differentiation of human teratocarcinoma NTERA cells.

**Fig. 1:**
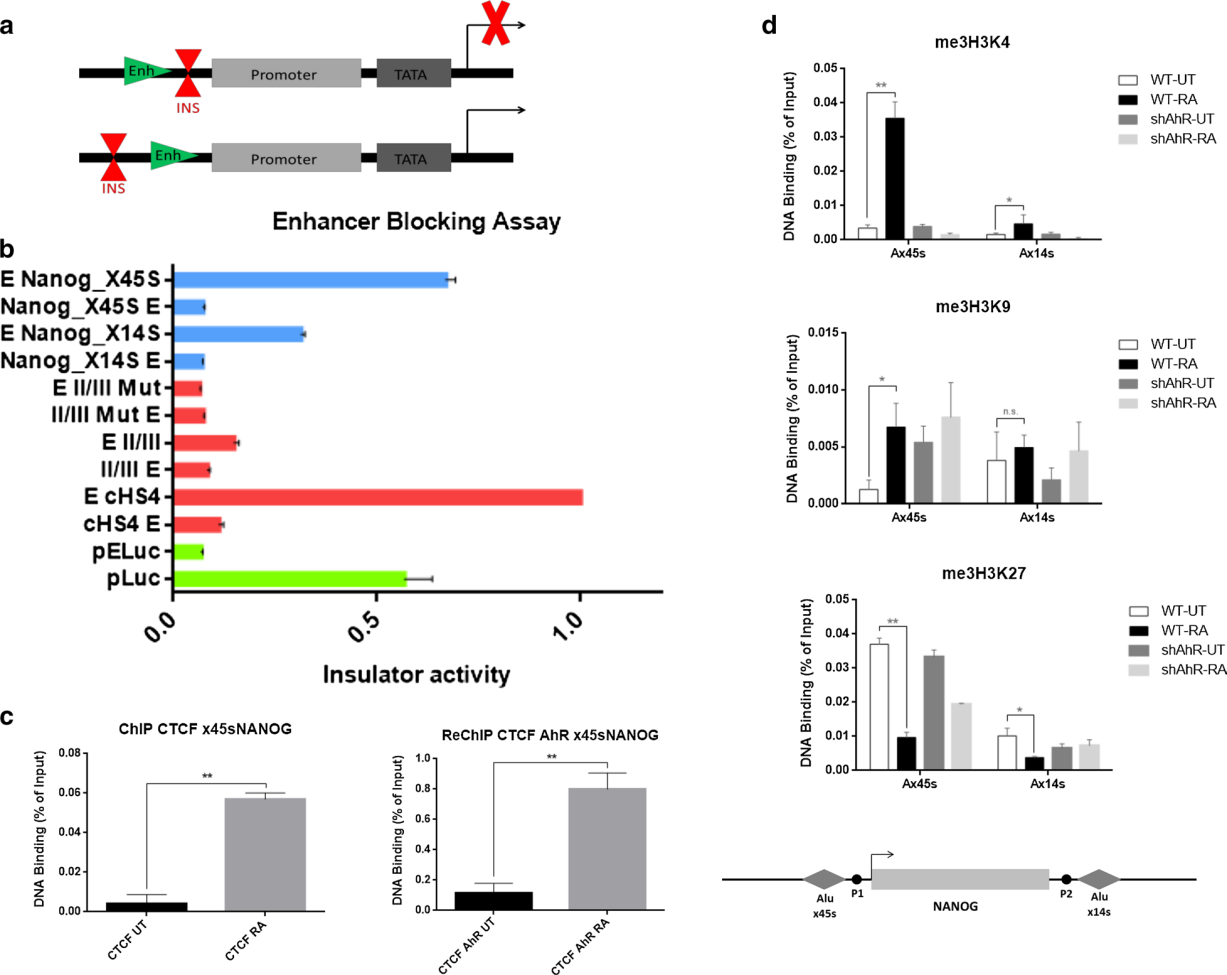
Analysis of enhancer-blocking activity and histone methylation marks of the Alu elements located flanking the Nanog locus. **a** Scheme of the enhancer-blocking assay (EBA). **b** Insulator activity of NANOG x45s and x14s Alu elements using human HEK293 cell line. Constructs (blue bars) were transiently transfected and their activity analyzed by EBA. Data are showed as fold-enhancer blocking activity normalized to the reference pELuc vector. **c** Chromatin immunoprecipitation (ChIP) and re-ChIP for CTCF binding to the Nanog x45s Alu were done in NTERA2-wt cells left untreated (UT) or treated with 1 µM of RA for 48 h. For specificity, one primer for the qPCR reaction to amplify each Alu was located in a unique genomic sequence flanking the transposon (see Additional file 3: Table S2). Re-ChIP involved a first immunoprecipitation with CTCF antibody followed by a second immunoprecipitation with AhR antibody. Input DNAs, immunoprecipitation without specific antibodies and immunoprecipitation with GAPDH antibody were also preformed. **d** Analysis of the pattern of histone methylation marks in the regions of the Alu elements x45s and x14s flanking *NANOG* locus in NTERA2-wt UT, RA for 48 h and NTERA2-sh UT, RA for 48 h. Three biological replicates and three experimental replicates were done for panel B. Three biological replicates and two experimental replicates were done for panels C and D. *P < 0.05, **P < 0.01 and ***P < 0.001. Data are shown as mean ± SD

Since murine B1 retrotransposons located in mouse gene promoters were able to act as genomic insulators after AhR binding [32], we first analyzed if the Alu elements that flanked the Nanog locus could also have insulator activity. As a read-out of this genomic function, we quantified the enhancer-blocking activity (EBA) of the X14S and X45S Alu elements using human HEK293 cells and a enhancer–promoter strategy previously described [32] (Fig. 1a). We found that, even when both X14S and X45S Alu elements showed insulator activity, the insulator effect of Nanog X45S was significantly more potent than that of X14S in HEK293 cells (Fig. 1b). Therefore, we next analyzed the role of the X45S element in RA-induced NTERA cell differentiation. Using chromatin immunoprecipitation (ChIP), we found that [26, 36] CTCF, a transcription factor frequently found in insulator and boundary elements, bound the Nanog X45S region upon cell differentiation but not under basal cell conditions (Fig. 1c, left). As AhR can also bind to the same genomic sequence after cell differentiation [17], we next studied if both AhR and CTCF were bound together. Using sequential double ChIP (re-ChIP), we observed strong co-recruitment of both proteins in differentiated NTERA cells as compared to basal undifferentiated conditions (Fig. 1c, right). These results suggest that the Nanog X45S element may act as genomic insulator preventing Nanog expression in differentiated NTERA cells.

### Histone methylation marks in the Nanog locus change after differentiation in an AhR-dependent manner

A common feature of most insulators is their ability to alter heterochromatin conformation, eventually resulting in the generation of epigenetic barriers [37–39]. We decided to test if differentiation could be associated to the formation of chromatin barriers in the Nanog locus by mapping three specific histone methylation marks using ChIP: trimethyl-H3K9 (3meH3K9), trimethyl-H3K4 (3meH3K4) and trimethyl-H3K27 (3meH3K27) (Fig. 1d). We found that 3meH3K4 levels were low in untreated NTERA cells along the Nanog locus and around the X14S and X45S elements (Fig. 1d, top, blue line). Cell differentiation provoked an increase in this epigenetic mark but only near the X45S element located next to the promoter region of Nanog (Fig. 1d, top, red line). Interestingly, AhR-silenced NTERA-2 cells previously developed [17] were insensitive to changes in 3meH3K4 after differentiation (Fig. 1d, top, green and black lines). In the case of the 3meH3K9 mark, we found low levels around the two Alu elements before and after cell differentiation, and AhR silencing did not significantly alter that result (Fig. 1d, center). Finally, 3meH3K27 levels were low around the downstream X14S element regardless of cell differentiation or AhR expression. Nevertheless, 3meH3K27 levels around the upstream X45S element were high in undifferentiated basal NTERA cells to become significantly reduced after RA-induced differentiation (Fig. 1d, bottom, blue and red lines). Interestingly, AhR silencing did not significantly affect the high basal levels of 3meH3K27 around X45S (Fig. 1d, bottom, green line), which remained insensitive upon differentiation (Fig. 1d, bottom, black line). Then we used the Epigenome Gateway webserver (http://epigenomegateway.wustl.edu) in order to explore these epigenetic marks within the Nanog locus in other published datasets. We found that consistent cell-type-specific changes of histone methylation patterns were observed at the X45S element when we compare hESC neuronal differentiation and RA-induced NTERA cell differentiation (Additional file 1: Figure S2). Altogether, these results indicated that the 3meH3K4 and 3meH3K27 epigenetic patterns surrounding the Alu elements located near Nanog can be relevant during stem cell differentiation, and that AhR silencing impaired those epigenetic changes only after NTERA cell differentiation.

### NTERA cell differentiation drives the formation of a chromatin loop between X45S and X14S Alus

**Fig. 2:**
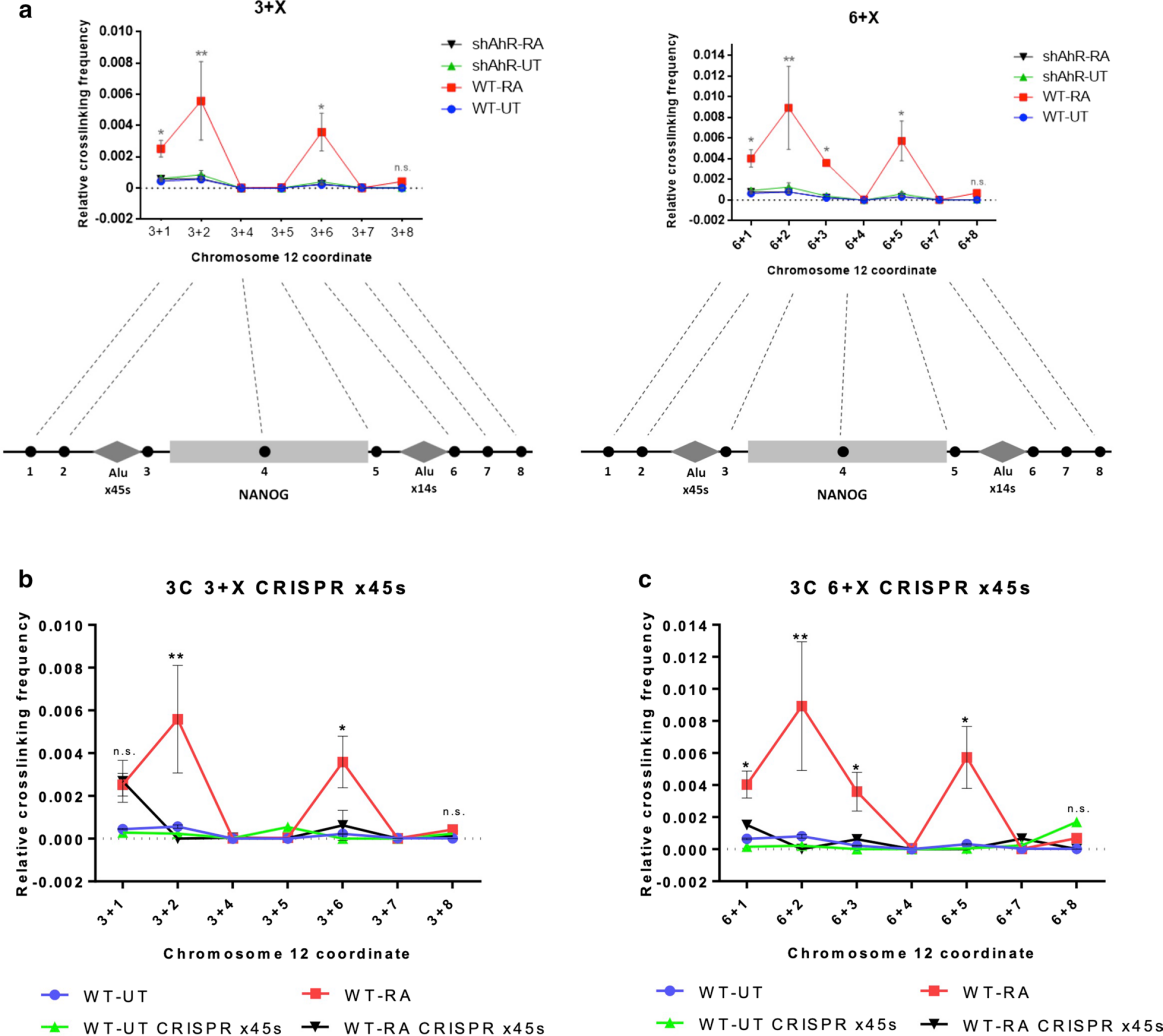
Human Nanog locus forms a chromatin loop between x45s and x14s Alus upon cell differentiation in NTERA cell line. **a** Chromosome conformation capture (3C) assay using coordinates 3 and 6 as hooks. The relative crosslinking frequency was quantified in NTERA-wt cells untreated (UT, blue), treated with RA for 48 h (red) and in NTERA-sh cells UT (green), RA for 48 h (black). 3 + X (left) and 6 + X (right) primer combinations were addressed. **b** Chromosome conformation capture (3C) assay using coordinate 3 as hook. The relative crosslinking frequency was quantified in NTERA-wt cells untreated (UT, blue), treated with RA for 48 h (red) and in NTERA-CRISPRx45s cells UT (green), RA for 48 h (black). 3 + X primers combinations were addressed. **c** 3C assay using coordinate 6 as hook. The relative crosslinking frequency was quantified in NTERA-wt cells untreated (UT, blue), treated with RA for 48 h (red) and in NTERA-CRISPRx45s cells UT (green), RA for 48 h (black). 6 + X primers combinations were addressed. Three biological replicates and two experimental replicates were done for **a**. Two biological replicates and two experimental replicates were done for **b** and **c**. *P < 0.05, **P < 0.01 and ***P < 0.001. Data are shown as mean ± SD

The epigenetic effects observed after NTERA cell differentiation led us to study the chromatin structure around the Nanog locus. We used 3C (chromosome conformation capture) assays to assess the presence of chromatin loops and TADs in the flanking regions of X14S and X45S Alus. Using a bait oligonucleotide located near the X45S retrotransposon (hook 3), we quantified the interaction between this Alu element and several other marks along the Nanog locus (3 + X, Fig. 2a, left). We did not find any chromatin loop formed in undifferentiated NTERA cells. Nevertheless, after cell differentiation, major interactions of X45S with two genomic regions appeared enriched: one upstream of X45S (mark 2) and another downstream of the X14S element (mark 6). Importantly, AhR silencing completely abolished the generation of these chromatin loops (Fig. 2a, left). In order to confirm these results, we performed 3C experiments using a bait oligonucleotide near the X14S retrotransposon (hook 6 + X, Fig. 2a, right). We again found an absence of chromatin loops in untreated undifferentiated NTERA cells and prominent interactions of X14S with two genomic regions following differentiation: one upstream of the X45S element (mark 2) and one more near the 3′ end of Nanog (mark 5). Consistently, generation of these chromatin interactions was dependent on the presence of AhR. Finally, we assessed the direct role of the X45S retrotransposon in the formation of the chromatin loops in the Nanog locus. The deletion of the Alu x45s element via CRISPR/Cas9 resulted in a loss of the interaction in most of the chromatin loop regions analyzed in differentiated N-TERA2 cell line (Fig. 2b, c). This effect was particularly pronounced in Alu x45s and Alu 14s loci (Fig. 2b and c, 3 + 6 primers combination). These results suggested that a chromatin loop flanking the Nanog gene was formed during NTERA cell differentiation and that such process likely involved the Alu retrotransposons and was AhR dependent.

**Fig. 3:**
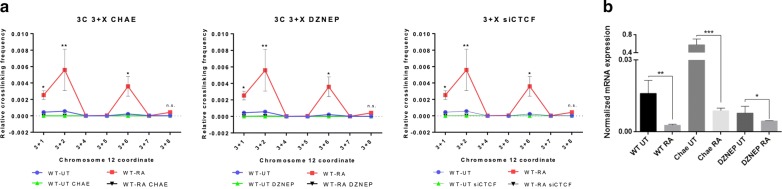
**a** 3C experiments with chaetocin (left) and deazaneplanocin-A (center) treatments, and CTCF siRNA transfection (right), in NTERA-wt UT (blue), treated with RA for 48 h (red), treated with chaetocin, deazaneplanocin-A or transfected with CTCF siRNA with or without RA (black and green, respectively). **b***NANOG* mRNAs were quantified by RT-qPCR in NTERA2 cell line left untreated (UT) or treated with 1 µM RA for 48 h and/or chaetocin/deazaneplanocin-A for 48 h. *GAPDH* mRNA was used to normalize gene expression (A Ct) and 2^−AACt^ to calculate variations with respect to control or untreated conditions. Three biological replicates and two experimental replicates were done for panels A. Four biological replicates and two experimental replicates were done for panel B. *P < 0.05, **P < 0.01 and ***P < 0.001. Data are shown as mean ± SD

We next assessed if the epigenetic modifications induced by cell differentiation in X45S could alter chromatin loop formation. Using chaetocin (Chae) and 3-deazaneplanocin-A (Dz), inhibitors of global histone methylation and H3K27-specific methylation, respectively, we found that both of these molecules significantly abrogated chromatin interactions between X45S and X14S Alu regions in differentiated NTERA cells (Fig. 3a, 3 + X; Additional file 1: Figure S3A, 6 + X). In addition, CTCF silencing also impaired the loop formation within the Nanog locus (Fig. 3a, 3 + X; Additional file 1: Figure S3A, 6 + X), supporting the idea of a epigenomic insulator associated to this chromatin loop. Moreover, Chae and Dz treatment also impaired histone methylation levels in NTERA cells as determined by immunoblotting (Additional file 1: Figure S3B). As Chae and Dz altered the epigenetic methylation profile and the chromatin architecture around the Nanog locus, we analyzed if these drugs could also affect Nanog expression. In agreement, we found that downregulation of Nanog in RA-differentiated cells was partially rescued by histone methylation inhibition using Chae (Fig. 3b), while basal levels of Nanog were affected by Chae and Dz. Thus, our data suggest that histone methylation was required for chromatin loop formation between flanking Alu retrotransposons that led to Nanog repression during NTERA cell differentiation.

### Engineered chromatin immunoprecipitation (enChIP) analysis identified target proteins that bind to the chromatin loop during cell differentiation

**Fig. 4:**
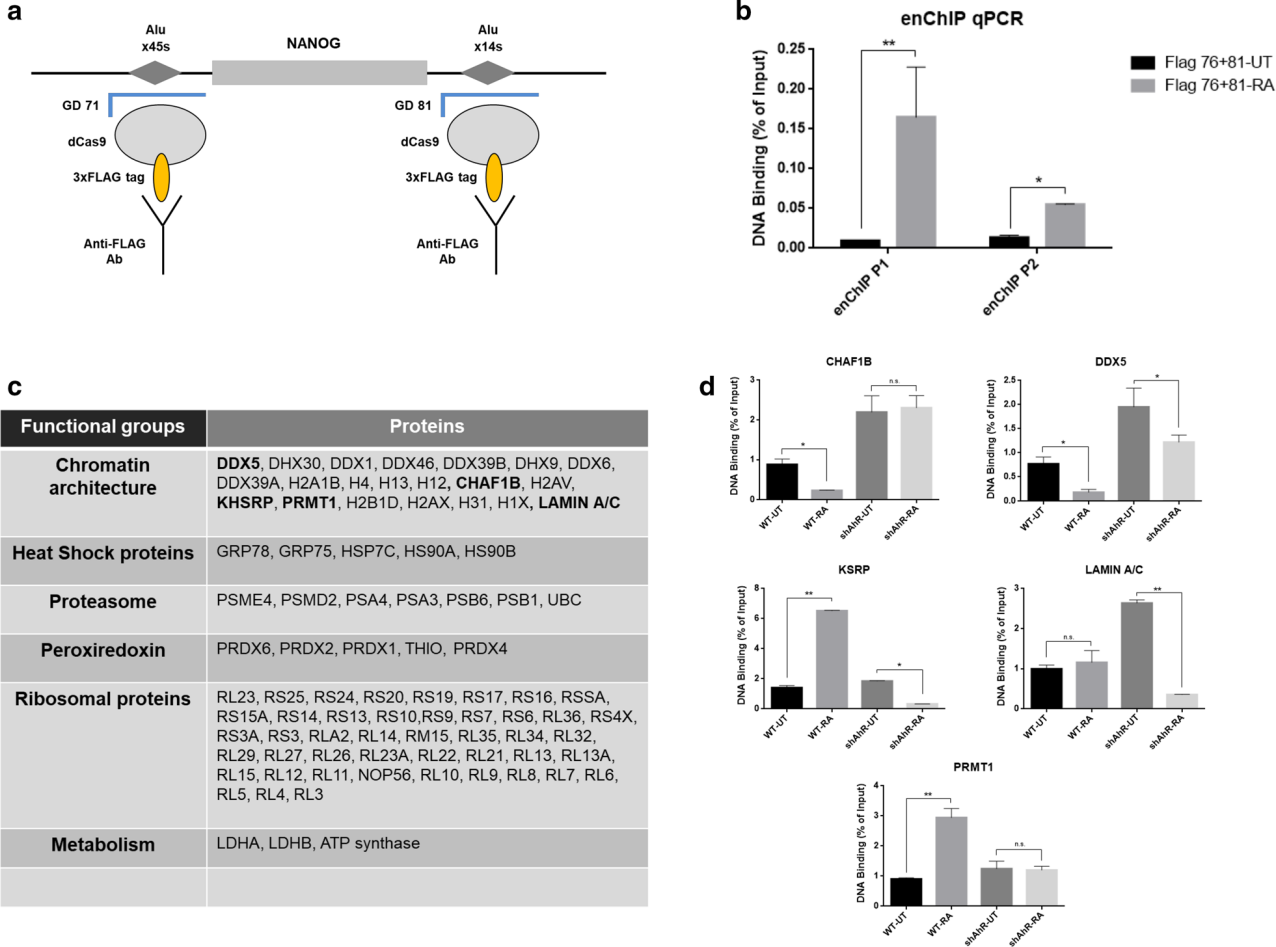
Dynamics of chromatin architecture-related proteins involved in the formation of Nanog chromatin loop during cell differentiation. **a** Scheme of the engineered chromatin immunoprecipitation (enChIP) 3× FLAG-dCas9 technique. **b** Chromatin immunoprecipitation (ChIP) for FLAG binding to the Nanog x45s and x14s Alus were done in NTERA2-wt cells left untreated (UT) or treated with 1 µM of RA for 48 h. ChIP was quantified by qPCR using specific oligonucleotides (see Additional file 3: Table S2). Input DNAs and immunoprecipitation without specifics antibodies were also preformed. **c** Table of main *NANOG* chromatin loop interacting proteins obtained with enChIP-dCas9 proteomic analysis (complete information enclosed in Additional file 3: Table S2). **d** Chromatin immunoprecipitation (ChIP) for CHAF1B, DDX5, KSRP, LAMIN A/C and PRMT1 binding to the Nanog x45s and x14s Alus were done in NTERA2-wt cells left untreated (UT) or treated with 1 µM of RA for 48 h. ChIP was quantified by qPCR using specific oligonucleotides (see Additional file 3: Table S2). Input DNAs and immunoprecipitation without specifics antibodies were also preformed for normalization and negative controls, respectively. Three biological replicates and three experimental replicates were done for panels B and D. *P < 0.05, **P < 0.01 and ***P < 0,001. Data are shown as mean ± SD

These results prompted us to study if additional proteins could be recruited for the formation of the chromatin loop around both Alu retrotransposons. To achieve this goal, we used a proteomic experimental approach based on the enChIP technology [40, 41] (see “Methods” section). Briefly, two genome-specific guide RNAs (gRNA) were designed to direct a Flag-tagged nuclease-dead Cas9 protein (dCas9) to X45S and X14S Alus (Fig. 4a and Additional file 1: Figure S4). We then used immunoprecipitation to retrieve the DNA–protein complexes captured by an anti-Flag antibody followed by dissociation of proteins from DNA. In this way, we could identify by proteomic analysis the specific set of proteins bound to the points of interaction generating the loop in the flanking regions of Nanog in differentiated NTERA cells. As a control of specificity, we confirmed by qPCR that the chromatin immunoprecipitated by the sgRNA-dCas9-anti-Flag antibody complex was specific for the Nanog locus and significantly enriched upon cell differentiation, which supports a change of local accessibility between the X45S and X14S Alus (Fig. 4b). Once isolated from DNA, proteins present in the interacting region were characterized by mass spectrometry (Fig. 4c and Additional file 2: Table S1). Identified proteins were classified in different functional groups shown in Fig. 4c. We focused on proteins involved in chromatin architecture and epigenetics and select five candidates (namely CHAF1B, DDX5, KSRP, LAMIN A/C and PRMT1) in order to study their recruitment dynamics to the interacting region during NTERA cell differentiation (Fig. 4d). For these experiments, we decided to use the X45S Alu since such upstream region was the one more significantly affected in its methylation status and in the generation of chromosomal interactions after differentiation. While CHAF1B and DDX5 were highly bound to this region under basal undifferentiated conditions, and such binding was decreased by differentiation, KSRP and PRMT1 showed an opposite pattern with increased binding to X45S after cell differentiation. Finally, LAMIN A/C relative binding to this region was unaffected by differentiation in NTERA cells. We then tested if AhR could contribute to these dynamic recruitments. Interestingly, we found that CHAF1B and DDX5 increased their binding to this region in the absence of AhR and that CHAF1B binding did not significantly respond to RA treatment in this AhR-defective condition. On the other hand, AhR deficiency did not affect basal KSRP and PRMT1 binding to the retrotransposon although it prevented its increase after RA-induced differentiation. LAMIN A/C recruitment to the X45S region was increased in AhR knockdown cells and significantly reduced upon RA treatment. These results indicated that AhR has an important role in the regulation of dynamic protein recruitment to the Nanog chromatin loop in differentiated cells. In addition, CHAF1B, DDX5, KSRP and PRMT1 might be also responsible for chromatin rearrangements in the vicinity of X45S and X14S retrotransposons as these factors were highly bound under basal undifferentiated (CHAF1B and DDX5) or differentiated (KSRP and PRMT1) conditions.

### CHAF1B and PRMT1 are required for chromatin loop formation in Nanog locus

**Fig. 5:**
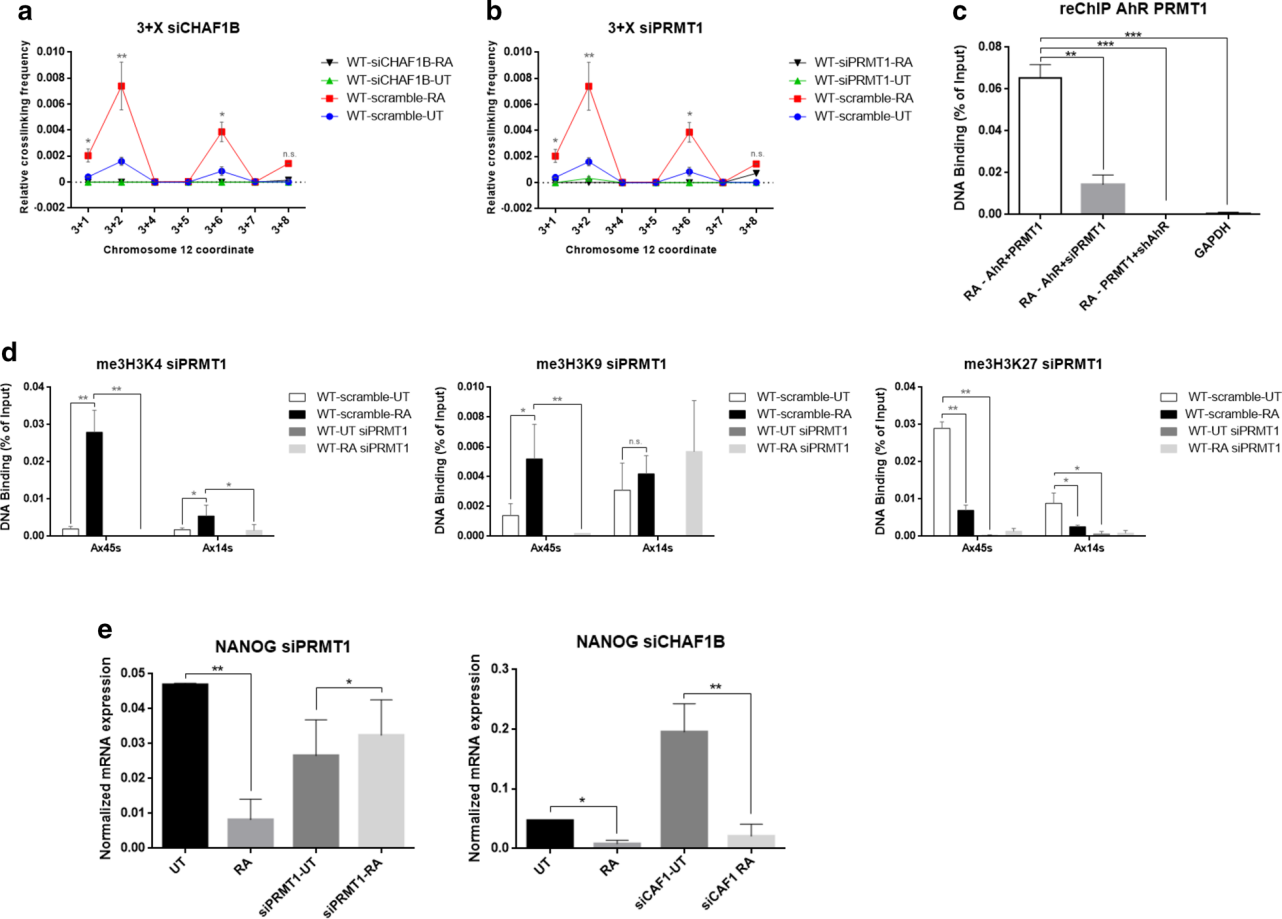
PRMT1 and CHAF1B drives the formation of *NANOG* locus Chromatin loop. **a** and **b** Chromosome Conformation capture (3C) assay using coordinate 3 as hook. The relative crosslinking frequency was quantified in NTERA-wt cells untreated (UT, blue), treated with RA for 48 h (red) and in NTERA-wt UT cells transfected with CHAF1B siRNA (*a*) or PRMT1 (*b*) (green), RA for 48 h (black). 3 + X primer combination was addressed. **c** Chromatin immunoprecipitation (ChIP) and re-ChIP for AhR and PRMT1 binding to the Nanog x45s Alu were done in NTERA2-wt cells treated with 1 µM of RA for 48 h. For specificity, one primer for the qPCR reaction to amplify each Alu was located in a unique genomic sequence flanking the transposon (see Additional file 3: Table S2). Re-ChIP involved a first immunoprecipitation with AhR antibody followed by a second immunoprecipitation with PRMT1 antibody. Input DNAs, immunoprecipitation without specifics antibodies and immunoprecipitation with GAPDH antibody were also preformed. **d** Analysis of the pattern of histone methylation marks in the regions of the Alu elements x45s and x14s flanking the Nanog locus in NTERA2-wt transfected with PRMT1 siRNA in conditions UT, RA for 48 h and NTERA2-sh UT, RA for 48 h. **e** Expression levels of *NANOG* mRNAs transfected with PRMT1 siRNA (left) or CHAF1B siRNA (right) were quantified by RT-qPCR in NTERA2 cell line left untreated (UT) or treated with 1 µM RA for 48 h. *GAPDH* mRNA was used to normalize gene expression (A Ct) and 2^−AACt^ to calculate variations with respect to control or untreated conditions. Three biological replicates and two experimental replicates were done for **a**, **b**, **c** and **d**. Three biological replicates and three experimental replicates were done for **e**. *P < 0.05, **P < 0.01 and ***P < 0.001. Data are shown as mean ± SD

We then selected two candidate proteins showing either increased (PRMT1) or decreased (CHAF1B) binding to the X45S region during NTERA cell differentiation. Using specific siRNAs, we silenced the expression of these two proteins (Additional file 1: Figure S5) in order to clarify their role in chromatin organization and in the transcriptional repression of Nanog during cell differentiation. Silencing of CHAF1B abolished loop formation in differentiated NTERA cells as determined by 3C assays (Fig. 5a). As CHAF1B was preferentially bound to retrotransposon regions prior to cell differentiation (Fig. 4d), it is possible that this protein is necessary but not sufficient for loop formation in undifferentiated NTERA cells. PRMT1 depletion also blocked chromatin interactions between the X45S and X14S regions under differentiating conditions (Fig. 5b), but since it was recruited mostly during cell differentiation, this protein may be needed to stabilize and/or maintain the chromatin loop. From previous studies [17], and from our current results, it seems that AhR and PRMT1 had a similar binding pattern to the X45S region in differentiated NTERA cells, and that their silencing abolished the reorganization of chromatin in the Nanog locus (Figs. 2a and 5b). In addition, PRMT1 binding to X45S was dependent on AhR expression (Fig. 4d). Therefore, we analyzed if AhR and PRMT1 were recruited together to the chromatin loop using ChIP and re-ChIP experiments (Fig. 5c). We found that, in differentiated NTERA cells, AhR and PRMT1 were bound together to the chromatin loop, and that either PRMT1 or AhR silencing reduced the interaction of both factors to that region. These data indicated that AhR and PRMT1 act in concert during the chromatin rearrangement that leads to Nanog repression upon cell differentiation. Consistent with this hypothesis, PRMT1 silencing produced similar effects than AhR silencing on the me3H3K4 epigenetic profile near the X45S and X14S Alu retrotransposons in differentiated NTERA cells (Fig. 5d). Specifically, 3meH3K4 levels around X45S became unresponsive to RA treatment in PRMT1-silenced cells (Fig. 5d) as they were in AhR-silenced cells (Fig. 1d). 3meH3K27 levels, on the contrary, were markedly depleted in PRMT1-silenced NTERA-2 cells (Fig. 5d) but not in AhR downregulated cells (Fig. 1d), suggesting that PRMT1 may influence 3meH3K27 levels in differentiated NTERA cells independently of AhR expression.

**Fig. 6:**
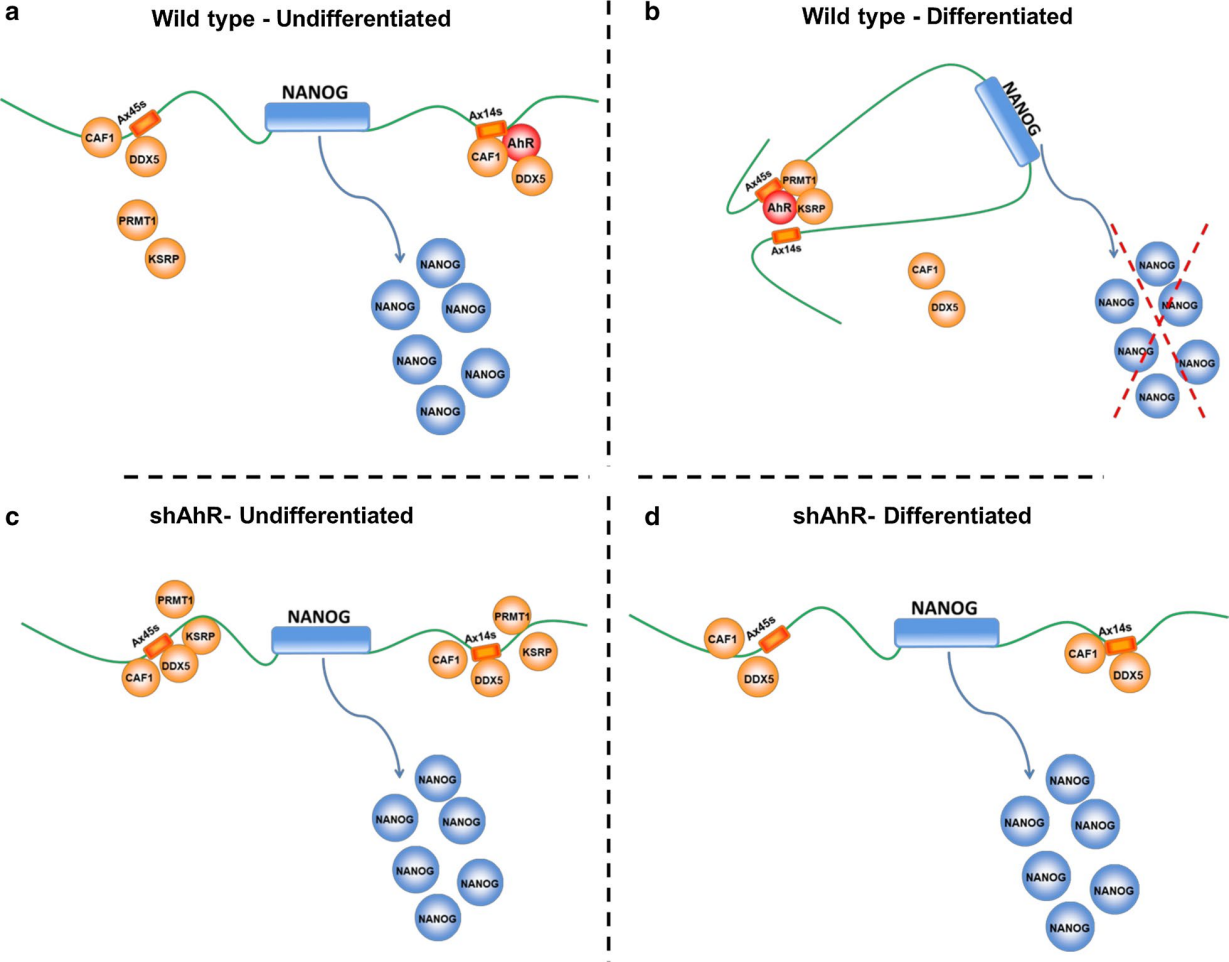
Scheme of proposed model of interaction between the protein complex and regulatory elements intervening in the regulation of *NANOG’*s expression in our model of carcinoma cell differentiation

Finally, we assessed if silencing of CHAF1B or PRMT1 could affect Nanog repression under differentiation (Fig. 5e). PRMT1 downmodulation induced a decrease in Nanog expression in basal NTERA cells that was not altered after differentiation. Furthermore, Nanog expression in CHAF1B-silenced cells was significantly higher under basal undifferentiated conditions compared to wild type, but RA treatment induced the decrease in Nanog expression. These results further supported the relevance of both PRMT1 and CHAF1B in regulating Nanog expression during differentiation. In summary, we describe a novel mechanism for the control of Nanog expression during differentiation of human teratocarcinoma cells (Fig. 6). Formation of a chromatin loop around Alu retrotransposons flanking the Nanog locus triggers a chromatin structure-driven process leading to its transcriptional repression. Such process is dependent on the transcription factor AhR while PRMT1 and CHAF1B seem to be convenient partners for chromatin loop formation and gene regulation.

## Discussion

In this paper, we describe the formation of a chromatin loop that encloses the Nanog locus during the differentiation of human teratocarcinoma NTERA cells. Such chromatin-dependent mechanism requires binding of a protein complex containing the aryl hydrocarbon receptor on Alu retrotransposons flanking Nanog and local changes in histone methylation. The main role of Alu retrotransposons in the control of gene expression through the generation of chromatin loops suggests that this type of regulatory mechanism might be observed in other genes or processes. Due to the wide presence of repetitive Alu elements in the human genome, these changes in chromatin architecture could become a common mechanism to downregulate or upregulate gene expression under different cell conditions. We have previously proposed that B1-SINE retrotransposons might act as genomic insulators defining gene expression domains as some of them have enhancer-blocking activity and bind well-known insulator proteins such as CTCF [31–33, 42]. Consistently, the Alu elements flanking Nanog also have enhancer-blocking activity and can bind CTCF in vivo. In addition, we have observed that other epigenomic datasets also present a differentiation-dependent epigenomic pattern in this locus. Our hypothesis could be extended to the dynamics of chromatin architecture since retrotransposon networks might dynamically act in the formation and disorganization of chromatin loops. Current and future studies aim to understand the signaling mechanisms that control the potential of thousands of repetitive elements to modulate gene expression. In this context, several groups, including ours, have described that repetitive elements provide binding sites for transcription factors whose recruitment will regulate gene expression. It is possible that the molecular mechanisms used for transcription factors to control gene expression differ depending on whether they are located in non-repetitive vs. repetitive genomic sequences.

We have used a proteomic screening derived from enChIP assays to generate candidate proteins involved in chromatin loop formation around Nanog locus. Bioinformatic analyses revealed that this screening favored the identification of structural proteins rather than transcription factors, but this is probably a consequence of the low number of transcription factor molecules assembled into the protein complex generating the loop. We have initially analyzed five candidates to then select two targets (PRMT1 and CHAF1B) for subsequent studies. Nevertheless, other identified proteins such as RNA-binding helicases may have a relevant role in inducing a chromatin reorganization to repress Nanog in differentiated cells, adding an additional level of complexity to the mechanism.

An important finding of this study is that an Alu retrotransposon may be required for the observed chromatin changes potentially insulating Nanog expression. In agreement, our previous work indicates that a SINE-B1 retrotransposon exerted an insulator effect to downregulate gene expression in mouse hepatocarcinoma cells [32, 35, 42], whereas transcription of the X45S Alu element was needed to repress Nanog in differentiated human teratocarcinoma NTERA cells [17]. Moreover, Nanog X45S and X14S Alu retroelements may be docking sites for proteins that participate in chromatin organization upon cell differentiation. Specifically, the arginine N-methyltransferase PRMT1, whose main target is histone H4 appears as a plausible candidate eventually modifying this non-canonical epigenetic mark. It is also possible that AhR may guide PRMT1 to the specific sites surrounding the Nanog locus that require arginine methylation. CHAF1B, on the other hand, is a component of the chromatin assembly factor 1 (CAF-I) necessary for chromatin assembly during DNA replication and repair. The fact that PRMT1 was recruited while CHAF1B was released from the chromatin loop suggests a role to promote loop formation for the former and an inhibitory function in loop generation for the second. Therefore, a combined mechanism of histone methylation and chromatin disassembly would be needed for loop formation and Nanog repression in differentiated cells. How these and other additional proteins, revealed by our proteomic analysis, participate in the control of Nanog during cell differentiation deserves further investigation. One example is Lamin A/C, detected in the enChIP experiment and whose recruitment to X45S was increased in AhR knockdown NTERA-2 cells and decreased after RA differentiation. A recent paper [43] shows several super-enhancer domains and TADs in the Nanog locus that can modulate its expression during cell differentiation, and Lamin A/C association to retrotransposons might affect the chromatin structure in this region. Another potential line of research would be to study if any of the proteins bound to the chromatin loop (Additional file 2: Table S1) are known to interact with Nanog or regulate its expression.

In conclusion, our work proposes the existence of a complex regulatory network of proteins involved in chromatin architecture and assembly, epigenetics and chromatin dynamics that control the formation of a chromatin loop between two Alu retrotransposons flanking the Nanog. As a consequence, Nanog expression can be downregulated during cell differentiation. Future studies will be needed to analyze if this loop is conserved in other physiological or pathological differentiating conditions.

## Methods

### Antibodies

The following antibodies were used: GAPDH (Cell signaling 2118, clone 14C10), NANOG (AbCam Ab-21624), AHR (ENZO Life Sciences BML-SA210), β-ACTIN (Sigma Aldrich A2066), β-TUBULIN (Thermo Scientific PA1-41331), Histone H3 (Upstate Millipore 06-755), me3H3K9 (Diagenode C15310013), me3H3K27 (Diagenode C15410069), CTCF (generous gift of Dr. Recillas-Targa), ANTI-FLAG M2 (Sigma-Aldrich F1804-200UG), PRMT1 (Bethyl A300-722A) and CHAF1B (Novus NB500-212).

### Cell lines and reagents

Human embryonic teratocarcinoma NTERA-wt and NTERA-sh cells were cultured in DMEM supplemented with 10% FBS, 100 µg/ml streptomycin, 100 U/ml penicillin and 2 mM l-glutamine at 37 °C with 5% CO_2_ atmosphere. Protein A/G-plus agarose was from Santa Cruz Biotechnology. The iScript™ Reverse transcription Supermix was from Bio-Rad (1708890) and the SYBR^®^ Select Master Mix was either from Life Technologies (4309155) or New England Biolabs (LUNA M3003L). Retroviral knockdown of human AHR in NTERA cell line was previously described (Morales-Hernández et al. [17]). The following siRNAs from Dharmacon were used: siGENOME CHAF1B (D-019937-01-0050), siGENOME PRMT1 (D-010102-01-0050), siGENOME CTCF siRNA (D-020165-03-0050) and siGENOME Non-Targeting siRNA (D-001206-14-05).

### Quantitative chromatin immunoprecipitation (qChIP)

Chromatin immunoprecipitation (ChIP) for AhR, CTCF, me3H3K4, me3H3K9, me3H3K27, PRMT1 and CHAF1B were performed in NTERA-wt cells essentially as described (Gómez-Durán et al. 2008, Román et al. [32]). NTERA-sh cells were used as negative controls. DNA was purified and eluted following manufacturer’s instructions using ChIP DNA Clean & Concentrator™ kit (Zymo Research D5205). Real-time PCR was done using SYBR^®^ Select Master Mix (Life Technologies) in a Step One Thermal Cycler (Applied Biosystems) as indicated (Morales-Hernández et al. [17]). Data are presented as percentage of DNA input in the antibody-containing immunoprecipitate minus the percentage of DNA input in the corresponding negative controls. The oligonucleotides used for ChIP are listed in Additional file 3: Table S2.

### Sequential ChIP (re-ChIP)

Sequential ChIP (re-ChIP) was performed as indicated [32]. GAPDH was used as a negative control for the second ChIP. Data are showed as percentage of DNA in the primary Immunoprecipitation. The primers used for re-ChIP are indicated in Additional file 3: Table S2.

### Enhancer-blocking assay

Enhancer-blocking assay was used to address the insulator activity of Nanog x45s and x14s Alu elements used the pELuc plasmid previously described (Lunyak et al. [30]). Alu elements were cloned between the CMV enhancer and the promoter or upstream of the CMV enhancer. The constructs were transfected into human embryonic HEK 293 cells as previously reported (Lunyak et al. [30]). Data were normalized as fold-enhancer blocking activity to the value achieved by the basal pELuc vector. Chicken 5′ HS4 beta-globin insulator element was used as positive control. The internal II/III boxes from the chicken 5′ HS4 beta-globin insulator element wild type and mutated were used as positive and negative controls, respectively [44].

### Bioinformatic analysis of Alu elements in stemness-related genes

The human genome was analyzed for the presence of conserved elements containing an XRE site and E-Box using an algorithm as previously described (Román et al. [35]). Alu elements were analyzed for their presence in 5′ and 3′ regions of pluripotency and stemness genes within a 200-bp interval from the transcription site.

### Chromosome conformation capture (3C)

Chromosome conformation capture (3C)-qPCR assay was performed as previously described [45]. Briefly, 1 × 10^7^ cells were cross-linked with formaldehyde and digested overnight with HindIII. Then, DNA was ligated overnight with T4 DNA Ligase (New England Biolabs M0202S) as previously described [45]. Subsequently, DNA was de-cross-linked overnight at 65 °C and purified by classic phenol–chloroform procedure. RT-qPCR was done with SYBR^®^ Select Master Mix (Life Technologies) in a Step One Real-Time PCR System (Life Technologies). The primer sequences used for quantification are listed in Additional file 3: Table S2. 3C-qPCR data were normalized using GAPDH as a loading control. The level of random collisions was normalized using the ubiquitously expressed ERCC3 locus.

### BAC preparation for 3C

BAC clones covering NANOG region of interest were prepared as previously described [45]. BAC clones were purified with NucleoBond^®^ BAC 100 kit (Macherey–Nagel 740579). An additional BAC clone was prepared covering the ERCC3 locus used as a control for qPCR normalization.

### CRISPR/Cas9 for Alu X45S

CRISPR/Cas9 experiments were performed as follows: N-TERA2 cells were transfected with 1000 ng of phCas9 plasmid and 600 ng of MLM3636-sgRNA plasmid used Lipofectamine 2000 (Life Technologies). Specific guided RNAs were designed targeting Alu x45s element: sgRNA-76_Fw ACACCCATCCTTAGTTGGCTGGGCGG and sgRNA-76_Rv AAAACCGCCCAGCCAACTAAGGATGG). Genomic DNA was obtained 72 h post-transfection with 500 µl/well of SDS-free buffer (50 mM KCl, 10 mM TrisHCl pH 8, 0.45% NP40, 0.45% Tween-20, 500 ng/ml Proteinase K), incubated 2 h at 56 °C and 15 min at 95 °C, and used directly for PCR. CRISPR activity was evaluated by T7 Endonuclease I assay or by PCR using primers designed to detect chromosomal deletions.

### enChIP-real-time PCR

Engineered chromatin immunoprecipitation (enChIP)-real-time PCR experiments were done as previously described (Fujita and Fujii [40]). Essentially, 5 × 10^6^ NTERA-wt were plated and transfected with 2 µg of 3× FLAGdCas9/pCMV-7.1 (Addgene) and 2 µg of sgRNA-NanogAlu 71/81 combination with Lipofectamine 3000 (Invitrogen). The specific guided RNAs sequences were (sgRNA-71_Fw ACACCGGCCGGGCTCCGTGGCTCATG, sgRNA-71_Rv AAAACATGAGCCACGGAGCCCGGCCG) and Alu X14S (sgRNA-81_Fw ACACCGATGGAGTCTCGCTCCTGTCG, sgRNA-81_Rv AAAACGACAGGAGCGAGACTCCATCG). After 48 h with or without 1 µM RA treatment, cells were fixed with 1% formaldehyde at RT for 15 min and quenched with glycine 0.125 M for 5 min. The chromatin fraction was extracted and sonicated as described in ChIP protocol. Subsequently, chromatin was pre-cleared with 120 µl of protein A/G-plus agarose (Santa Cruz Biotechnology) and incubated with 5 µg of anti-FLAG M2 antibody (Sigma-Aldrich) and 120 µl of protein A/G-plus agarose at 4 °C overnight. The samples were washed, de-cross-linked and DNA was purified as previously indicated in ChIP protocol. The primers used for real-time PCR are listed in Additional file 2: Table S2. 3× FLAG-dCas9/pCMV-7.1 was a gift from Hodaka Fujii (Addgene plasmid# 47948).

### enChIP-mass spectrometry (MS)

For the enChIP-MS experiment, cells were plated, treated and transfected as previously indicated in enChIP-real-time PCR protocol. Then, cells were lysed and prepared for SDS-PAGE analysis following the manufacturer’s recommendations using the Miltenyi’s µMACS DYKDDDDK Isolation Kit (130-101-591). The proteins were visualized by silver staining. Protein bands were excised and analyzed using mass spectrometry in the Cancer Research Center proteomics facility.

### SDS-PAGE and immunoblotting

Nuclear and cytosolic cellular extracts were prepared for NTERA-wt and NTERA-sh as described previously [46]. SDS-PAGE and western blotting for both cell lines were performed as indicated [47]. In brief, aliquots of 25 µg total protein was electrophoresed in 7.5% SDS-PAGE gels and transferred to nitrocellulose membranes by electroblotting. Then, membranes were blocked in TBS-T solution containing 5% non-fat milk and sequentially incubated with primary and secondary antibodies, washed in TBS-T and revealed using Clarity™ Western ECL Substrate (Bio-Rad 1705060). Quantification of protein expression was done in a ChemiDoc XRS + equipment (Bio-Rad).

### Reverse transcription and real-time PCR

Total RNA was isolated following manufacturer’s indications using the High Pure RNA Isolation Kit (Roche 11828665001). Reverse transcription was performed using the iScript™ Reverse transcription Supermix (Bio-Rad). Real-time PCR was done using SYBR^®^ Select Master Mix (Life Technologies) or LUNA (New England Biolabs) in a Step One Thermal Cycler (Applied Biosystems) as described (Morales-Hernández et al. [17]). The primers used for RT-qPCR ar indicated in Supplementary Table 2 (S2).

### Statistical analyses

Comparisons between experimental conditions were done using GraphPad Prism 6.0 software (GraphPad). The unpaired two-sided Student’s *t* test was used to analyze differences between two experimental groups. Analyses of three or more groups were addressed using ANOVA. The Mann–Whitney non-parametric statistical method was used for comparisons of rank variations between independent groups. Data are shown as mean ± SD. Significant differences were considered at *P < 0.05, ** *P* < 0.01, *** *P* < 0.001.

## Supplementary information

**Additional file 1.** Additional figures of the manuscript including supporting information.

**Additional file 2: Table S1.** Complete list of genes encoding identified proteins bound to the X45S and X14S Alu loci obtained via enChIP-mass spectrometry in N-TERA2 cell line.

**Additional file 3: Table S3.** Complete list of primers used in chIP, 3C, enchIP and CRISPR experiments.

## Data Availability

The datasets used during the current study, including cell lines and plasmids are available from the corresponding author on reasonable request.
