# Supplementary figures and images for: Alu retrotransposons modulate Nanog expression through dynamic changes in regional chromatin conformation via aryl hydrocarbon receptor
Source: Epigenetics Chromatin. 2020 Mar 14;13:15. doi: 10.1186/s13072-020-00336-w (PMC7071633; doi:10.1186/s13072-020-00336-w)

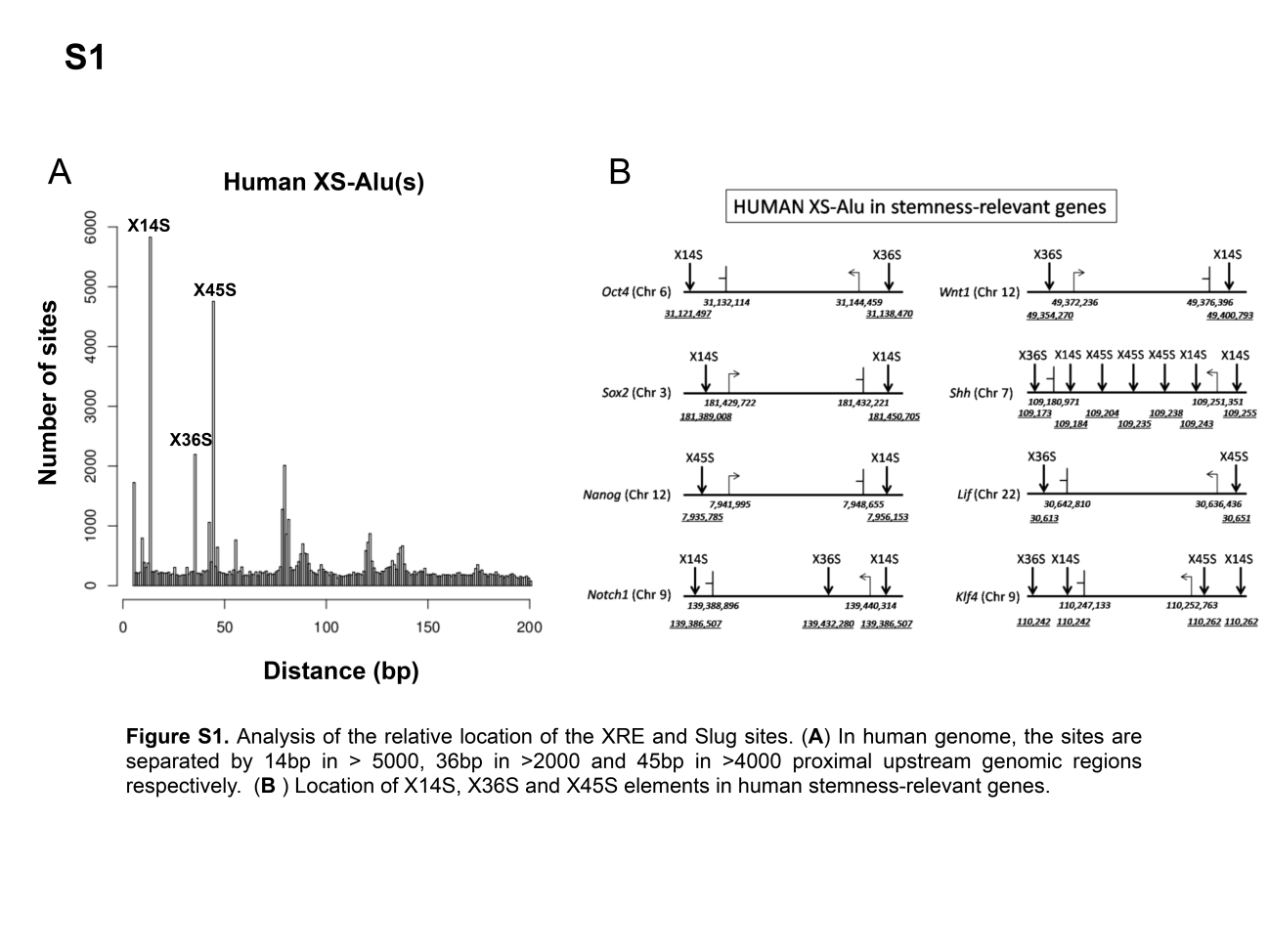


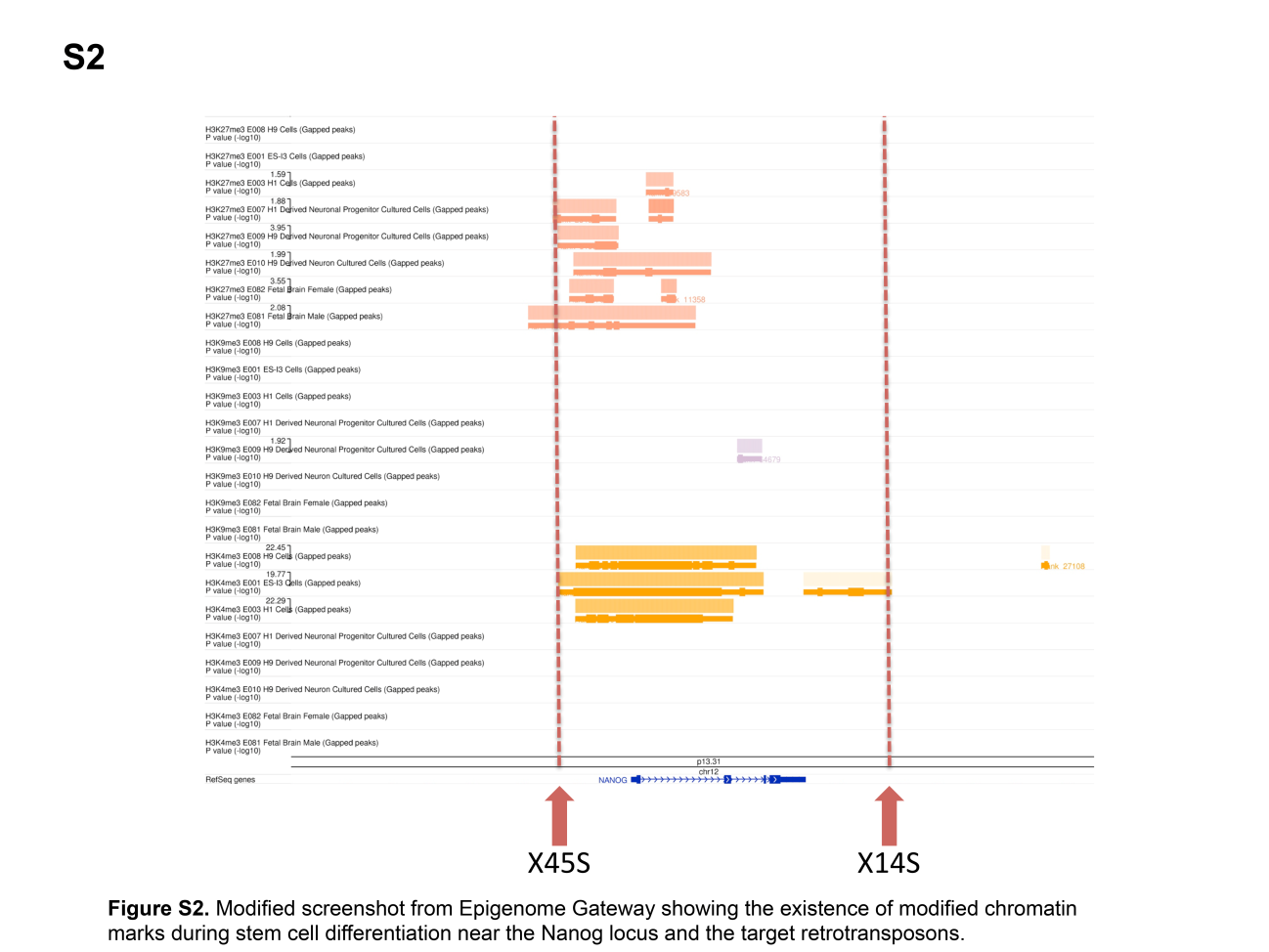


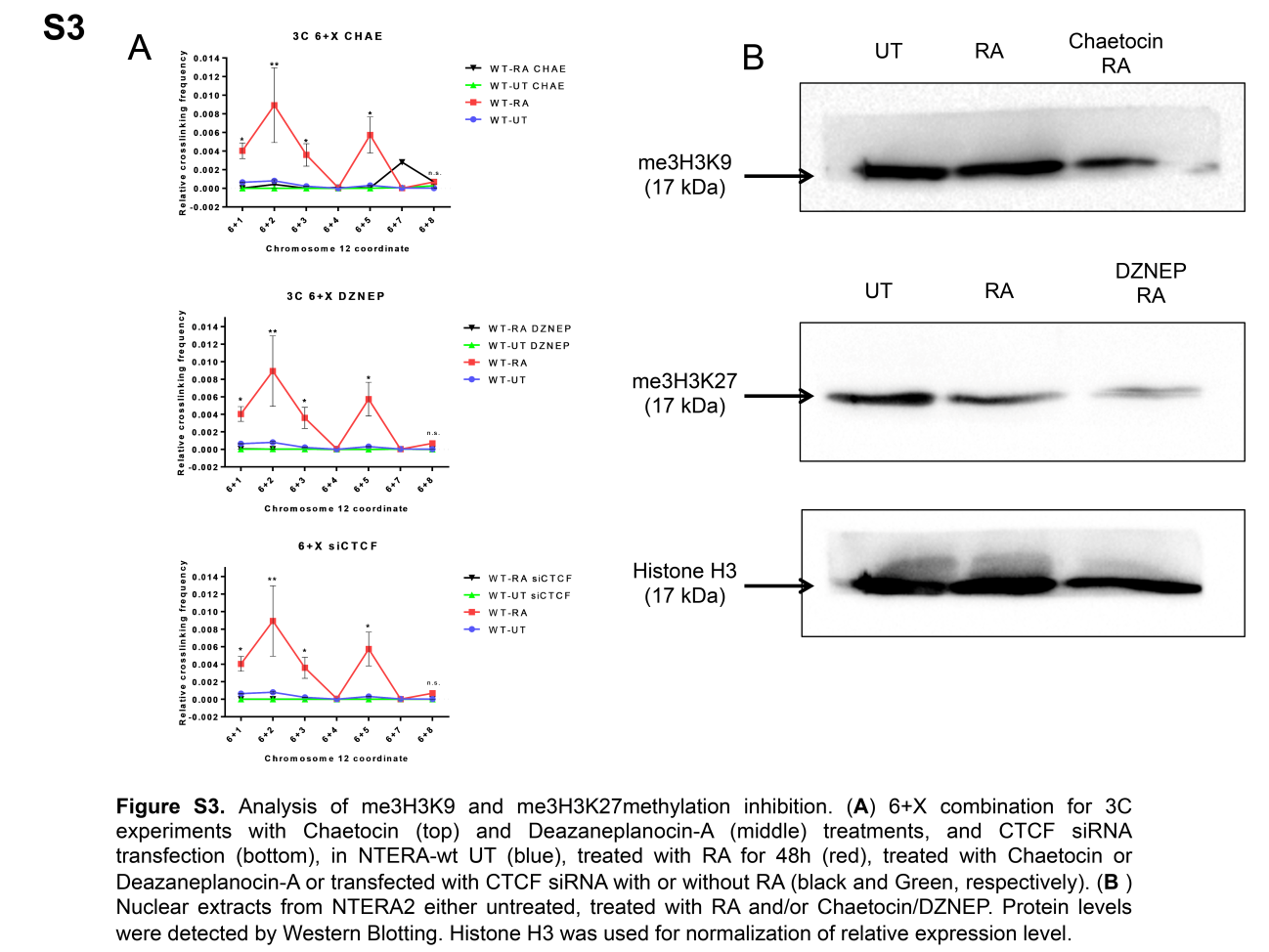


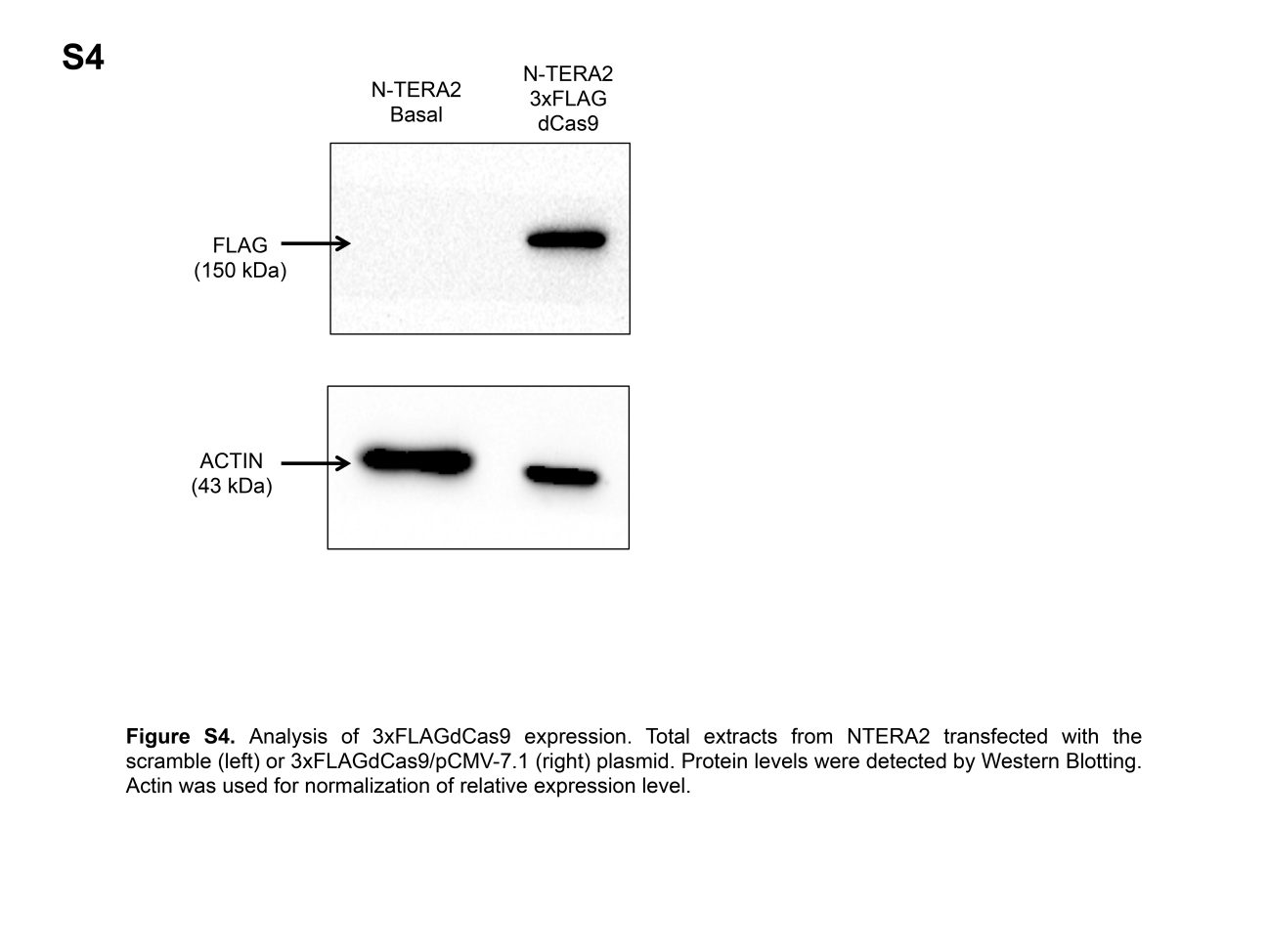


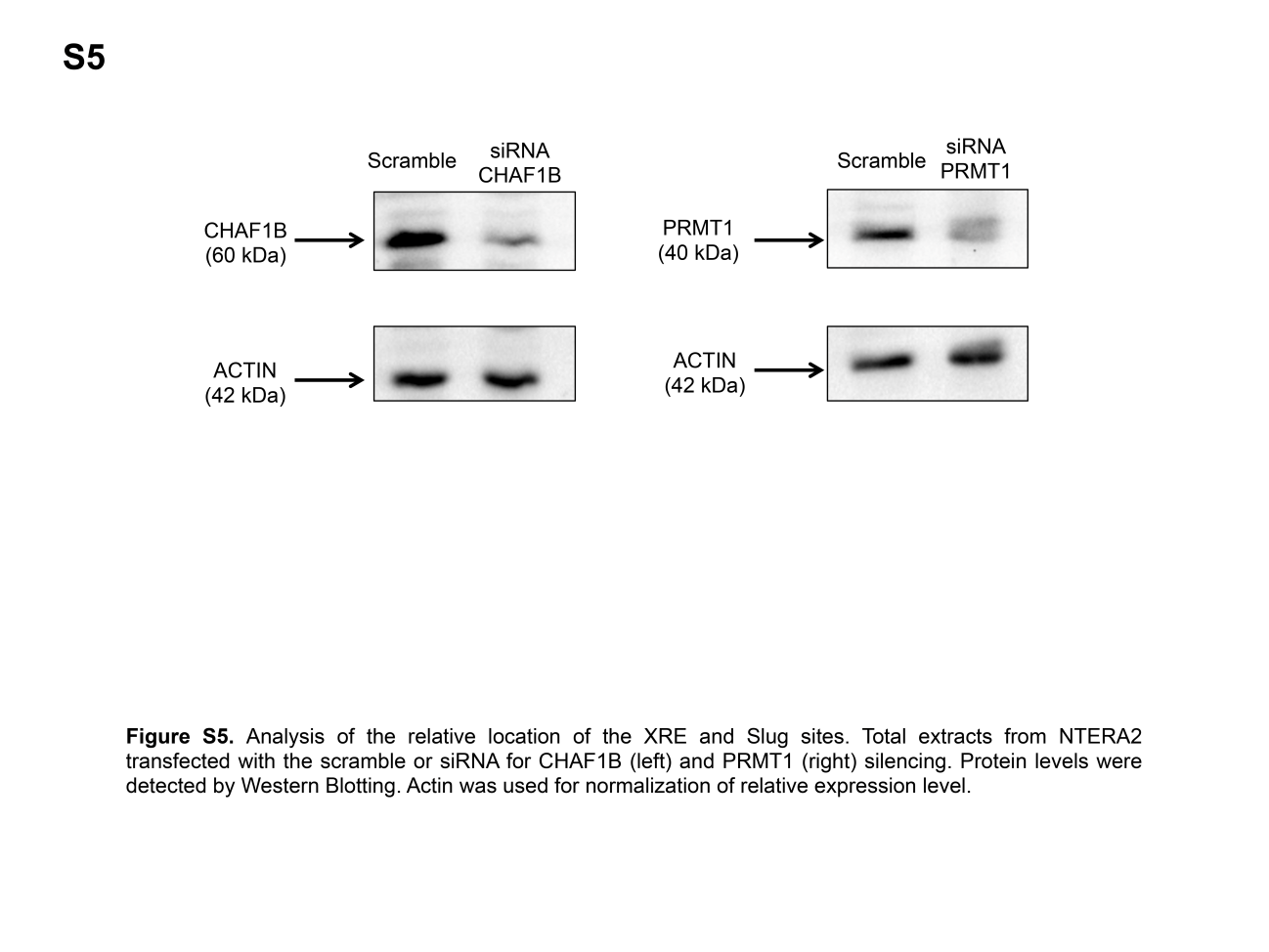

Supplement: Supplementary file 1 — Additional file 1. Additional figures of the manuscript including supporting information. [file 13072_2020_336_MOESM1_ESM.docx]
