# Supplementary material for: Alu retrotransposons modulate Nanog expression through dynamic changes in regional chromatin conformation via aryl hydrocarbon receptor
Source: Epigenetics Chromatin. 2020 Mar 14;13:15. doi: 10.1186/s13072-020-00336-w (PMC7071633; doi:10.1186/s13072-020-00336-w)
Supplement: Supplementary file 2 — Additional file 2: Table S1. Complete list of genes encoding identified proteins bound to the X45S and X14S Alu loci obtained via enChIP-mass spectrometry in N-TERA2 cell line. [file 13072_2020_336_MOESM2_ESM.docx]

**Table S1.** Complete list of genes encoding identified proteins bound to the X45S and X14S Alu loci obtained via enChIP-Mass Spectrometry in N-TERA2 cell line.

| **Protein** | **Gene name** |
| --- | --- |
| Ig kappa chain C region OS=Homo sapiens GN=IGKC PE=1 SV=1 - | IGKC |
| Desmoglein-1 OS=Homo sapiens GN=DSG1 PE=1 SV=2 - | DSG1 |
| Glyceraldehyde-3-phosphate dehydrogenase OS=Homo sapiens GN=GAPDH PE=1 SV=3 - | G3P |
| Immunoglobulin kappa variable 2D-29 OS=Homo sapiens GN=IGKV2D-29 PE=3 SV=1 - | KVD29 |
| ATP-citrate synthase OS=Homo sapiens GN=ACLY PE=1 SV=3 - | ACLY |
| Probable C-mannosyltransferase DPY19L1 OS=Homo sapiens GN=DPY19L1 PE=2 SV=1 - | D19L1 |
| Elongation factor 2 OS=Homo sapiens GN=EEF2 PE=1 SV=4 - | EF2 |
| Heat shock cognate 71 kDa protein OS=Homo sapiens GN=HSPA8 PE=1 SV=1 - | HSP7C |
| Isoform 2 of Voltage-dependent anion-selective channel protein 2 OS=Homo sapiens GN=VDAC2 - | VDAC2 |
| Isoform 2 of Heterogeneous nuclear ribonucleoprotein A1 OS=Homo sapiens GN=HNRNPA1 - | ROA1 |
| Tubulin beta-3 chain OS=Homo sapiens GN=TUBB3 PE=1 SV=2 - | TBB3 |
| Isoform 1 of Plakophilin-1 OS=Homo sapiens GN=PKP1 - | PKP1 |
| Tubulin beta-4B chain OS=Homo sapiens GN=TUBB4B PE=1 SV=1 - | TBB4B |
| Transketolase OS=Homo sapiens GN=TKT PE=1 SV=3 - | TKT |
| Nuclease-sensitive element-binding protein 1 OS=Homo sapiens GN=YBX1 PE=1 SV=3 - | YBOX1 |
| Catalase OS=Homo sapiens GN=CAT PE=1 SV=3 - | CATA |
| Histone H2B type 1-D OS=Homo sapiens GN=HIST1H2BD PE=1 SV=2 - | H2B1D |
| Leucine-rich PPR motif-containing protein, mitochondrial OS=Homo sapiens GN=LRPPRC PE=1 SV=3 - | LPPRC |
| Histone H1.2 OS=Homo sapiens GN=HIST1H1C PE=1 SV=2 - | H12 |
| Histone H1.3 OS=Homo sapiens GN=HIST1H1D PE=1 SV=2 - | H13 |
| Protein phosphatase 1B OS=Homo sapiens GN=PPM1B PE=1 SV=1 - | PPM1B |
| 40S ribosomal protein S3 OS=Homo sapiens GN=RPS3 PE=1 SV=2 - | RS3 |
| 60S ribosomal protein L13 OS=Homo sapiens GN=RPL13 PE=1 SV=4 - | RL13 |
| Suprabasin OS=Homo sapiens GN=SBSN PE=1 SV=2 - | SBSN |
| Histone H4 OS=Homo sapiens GN=HIST1H4A PE=1 SV=2 - | H4 |
| Heterogeneous nuclear ribonucleoproteins A2/B1 OS=Homo sapiens GN=HNRNPA2B1 PE=1 SV=2 - | ROA2 |
| Glycerol-3-phosphate dehydrogenase, mitochondrial OS=Homo sapiens GN=GPD2 PE=1 SV=3 - | GPDM |
| Zinc-alpha-2-glycoprotein OS=Homo sapiens GN=AZGP1 PE=1 SV=2 - | ZA2G |
| Serine/threonine-protein kinase 38 OS=Homo sapiens GN=STK38 PE=1 SV=1 - | STK38 |
| Peroxiredoxin-1 OS=Homo sapiens GN=PRDX1 PE=1 SV=1 - | PRDX1 |
| Cytosolic purine 5'-nucleotidase OS=Homo sapiens GN=NT5C2 PE=1 SV=1 - | 5NTC |
| Isoform 2 of 40S ribosomal protein S24 OS=Homo sapiens GN=RPS24 - | RS24 |
| 60S ribosomal protein L21 OS=Homo sapiens GN=RPL21 PE=1 SV=2 - | RL21 |
| 40S ribosomal protein S25 OS=Homo sapiens GN=RPS25 PE=1 SV=1 - | RS25 |
| Isoform 2 of Stomatin-like protein 2, mitochondrial OS=Homo sapiens GN=STOML2 - | STML2 |
| 60S ribosomal protein L11 OS=Homo sapiens GN=RPL11 PE=1 SV=2 - | RL11 |
| Dermcidin OS=Homo sapiens GN=DCD PE=1 SV=2 - | DCD |
| Heat shock protein HSP 90-beta OS=Homo sapiens GN=HSP90AB1 PE=1 SV=4 - | HS90B |
| Spliceosome RNA helicase DDX39B OS=Homo sapiens GN=DDX39B PE=1 SV=1 - | DX39B |
| Polyribonucleotide nucleotidyltransferase 1, mitochondrial OS=Homo sapiens GN=PNPT1 PE=1 SV=2 - | PNPT1 |
| 60S ribosomal protein L6 OS=Homo sapiens GN=RPL6 PE=1 SV=3 - | RL6 |
| Isoform 2 of ATP synthase subunit alpha, mitochondrial OS=Homo sapiens GN=ATP5A1 - | ATPA |
| Pyruvate kinase PKM OS=Homo sapiens GN=PKM PE=1 SV=4 - | KPYM |
| ATP-dependent RNA helicase DDX39A OS=Homo sapiens GN=DDX39A PE=1 SV=2 - | DX39A |
| 60S ribosomal protein L15 OS=Homo sapiens GN=RPL15 PE=1 SV=2 - | RL15 |
| Heat shock protein HSP 90-alpha OS=Homo sapiens GN=HSP90AA1 PE=1 SV=5 - | HS90A |
| L-lactate dehydrogenase B chain OS=Homo sapiens GN=LDHB PE=1 SV=2 - | LDHB |
| Isoform C of Prelamin-A/C OS=Homo sapiens GN=LMNA - | LMNA |
| Histone H2A type 1-B/E OS=Homo sapiens GN=HIST1H2AB PE=1 SV=2 - | H2A1B |
| ADP/ATP translocase 2 OS=Homo sapiens GN=SLC25A5 PE=1 SV=7 - | ADT2 |
| 60S ribosomal protein L3 OS=Homo sapiens GN=RPL3 PE=1 SV=2 - | RL3 |
| ATP-dependent RNA helicase A OS=Homo sapiens GN=DHX9 PE=1 SV=4 - | DHX9 |
| 60S ribosomal protein L12 OS=Homo sapiens GN=RPL12 PE=1 SV=1 - | RL12 |
| 60S ribosomal protein L13a OS=Homo sapiens GN=RPL13A PE=1 SV=2 - | RL13A |
| 40S ribosomal protein S3a OS=Homo sapiens GN=RPS3A PE=1 SV=2 - | RS3A |
| Thioredoxin OS=Homo sapiens GN=TXN PE=1 SV=3 - | THIO |
| Isoform 4 of Mediator of RNA polymerase II transcription subunit 23 OS=Homo sapiens GN=MED23 - | MED23 |
| 78 kDa glucose-regulated protein OS=Homo sapiens GN=HSPA5 PE=1 SV=2 - | GRP78 |
| Malate dehydrogenase, mitochondrial OS=Homo sapiens GN=MDH2 PE=1 SV=3 - | MDHM |
| Ras-related protein Rab-11B OS=Homo sapiens GN=RAB11B PE=1 SV=4 - | RB11B |
| 40S ribosomal protein S9 OS=Homo sapiens GN=RPS9 PE=1 SV=3 - | RS9 |
| 40S ribosomal protein S17 OS=Homo sapiens GN=RPS17 PE=1 SV=2 - | RS17 |
| Peroxiredoxin-2 OS=Homo sapiens GN=PRDX2 PE=1 SV=5 - | PRDX2 |
| Interleukin enhancer-binding factor 2 OS=Homo sapiens GN=ILF2 PE=1 SV=2 - | ILF2 |
| 40S ribosomal protein S13 OS=Homo sapiens GN=RPS13 PE=1 SV=2 - | RS13 |
| Heterogeneous nuclear ribonucleoprotein H OS=Homo sapiens GN=HNRNPH1 PE=1 SV=4 - | HNRH1 |
| Peroxiredoxin-4 OS=Homo sapiens GN=PRDX4 PE=1 SV=1 - | PRDX4 |
| Isoform 4 of Heterogeneous nuclear ribonucleoproteins C1/C2 OS=Homo sapiens GN=HNRNPC - | HNRPC |
| Isoform 4 of Heterogeneous nuclear ribonucleoprotein D0 OS=Homo sapiens GN=HNRNPD - | HNRPD |
| Insulin-like growth factor 2 mRNA-binding protein 1 OS=Homo sapiens GN=IGF2BP1 PE=1 SV=2 - | IF2B1 |
| 40S ribosomal protein S4, X isoform OS=Homo sapiens GN=RPS4X PE=1 SV=2 - | RS4X |
| 40S ribosomal protein S7 OS=Homo sapiens GN=RPS7 PE=1 SV=1 - | RS7 |
| ADP-ribosylation factor 4 OS=Homo sapiens GN=ARF4 PE=1 SV=3 - | ARF4 |
| Calmodulin-like protein 5 OS=Homo sapiens GN=CALML5 PE=1 SV=2 - | CALL5 |
| Neutrophil defensin 1 OS=Homo sapiens GN=DEFA1 PE=1 SV=1 - | DEF1 |
| 60S ribosomal protein L14 OS=Homo sapiens GN=RPL14 PE=1 SV=4 - | RL14 |
| 60S ribosomal protein L27 OS=Homo sapiens GN=RPL27 PE=1 SV=2 - | RL27 |
| Isoform 3 of Heterogeneous nuclear ribonucleoprotein A/B OS=Homo sapiens GN=HNRNPAB - | ROAA |
| 60S ribosomal protein L22 OS=Homo sapiens GN=RPL22 PE=1 SV=2 - | RL22 |
| L-lactate dehydrogenase A chain OS=Homo sapiens GN=LDHA PE=1 SV=2 - | LDHA |
| 60S ribosomal protein L8 OS=Homo sapiens GN=RPL8 PE=1 SV=2 - | RL8 |
| 40S ribosomal protein S19 OS=Homo sapiens GN=RPS19 PE=1 SV=2 - | RS19 |
| 60S ribosomal protein L7 OS=Homo sapiens GN=RPL7 PE=1 SV=1 - | RL7 |
| Heat shock protein beta-1 OS=Homo sapiens GN=HSPB1 PE=1 SV=2 - | HSPB1 |
| Bleomycin hydrolase OS=Homo sapiens GN=BLMH PE=1 SV=1 - | BLMH |
| 60S ribosomal protein L32 OS=Homo sapiens GN=RPL32 PE=1 SV=2 - | RL32 |
| 60 kDa heat shock protein, mitochondrial OS=Homo sapiens GN=HSPD1 PE=1 SV=2 - | CH60 |
| Polyubiquitin-C OS=Homo sapiens GN=UBC PE=1 SV=3 - | UBC |
| Far upstream element-binding protein 1 OS=Homo sapiens GN=FUBP1 PE=1 SV=3 - | FUBP1 |
| 60S ribosomal protein L36 OS=Homo sapiens GN=RPL36 PE=1 SV=3 - | RL36 |
| 40S ribosomal protein S20 OS=Homo sapiens GN=RPS20 PE=1 SV=1 - | RS20 |
| 60S ribosomal protein L29 OS=Homo sapiens GN=RPL29 PE=1 SV=2 - | RL29 |
| Vimentin OS=Homo sapiens GN=VIM PE=1 SV=4 - | VIME |
| Ubiquitin-conjugating enzyme E2 N OS=Homo sapiens GN=UBE2N PE=1 SV=1 - | UBE2N |
| ADP/ATP translocase 3 OS=Homo sapiens GN=SLC25A6 PE=1 SV=4 - | ADT3 |
| Isoform 2 of Inactive hydroxysteroid dehydrogenase-like protein 1 OS=Homo sapiens GN=HSDL1 - | HSDL1 |
| Mitochondrial carrier homolog 2 OS=Homo sapiens GN=MTCH2 PE=1 SV=1 - | MTCH2 |
| Isoform 2 of Triosephosphate isomerase OS=Homo sapiens GN=TPI1 - | TPIS |
| 40S ribosomal protein S10 OS=Homo sapiens GN=RPS10 PE=1 SV=1 - | RS10 |
| 40S ribosomal protein SA OS=Homo sapiens GN=RPSA PE=1 SV=4 - | RSSA |
| Isoform 2 of Small nuclear ribonucleoprotein Sm D3 OS=Homo sapiens GN=SNRPD3 - | SMD3 |
| Splicing factor 3B subunit 3 OS=Homo sapiens GN=SF3B3 PE=1 SV=4 - | SF3B3 |
| 60S ribosomal protein L23 OS=Homo sapiens GN=RPL23 PE=1 SV=1 - | RL23 |
| TREMBL:Q3SX09 (Bos taurus) similar to HBG protein |  |
| 60S ribosomal protein L9 OS=Homo sapiens GN=RPL9 PE=1 SV=1 - | RL9 |
| Eukaryotic initiation factor 4A-I OS=Homo sapiens GN=EIF4A1 PE=1 SV=1 - | IF4A1 |
| ATP synthase subunit beta, mitochondrial OS=Homo sapiens GN=ATP5B PE=1 SV=3 - | ATPB |
| GTP-binding nuclear protein Ran OS=Homo sapiens GN=RAN PE=1 SV=3 - | RAN |
| Isoform 2 of Filamin-C OS=Homo sapiens GN=FLNC - | FLNC |
| Isoform 2 of Probable ATP-dependent RNA helicase DDX5 OS=Homo sapiens GN=DDX5 - | DDX5 |
| Skin-specific protein 32 OS=Homo sapiens GN=XP32 PE=1 SV=1 - | XP32 |
| Isoform 3 of Putative ATP-dependent RNA helicase DHX30 OS=Homo sapiens GN=DHX30 - | DHX30 |
| Stonin-2 OS=Homo sapiens GN=STON2 PE=1 SV=1 - | STON2 |
| 40S ribosomal protein S15a OS=Homo sapiens GN=RPS15A PE=1 SV=2 - | RS15A |
| Peroxiredoxin-6 OS=Homo sapiens GN=PRDX6 PE=1 SV=3 - | PRDX6 |
| Lysosome-associated membrane glycoprotein 2 OS=Homo sapiens GN=LAMP2 PE=1 SV=2 - | LAMP2 |
| Eukaryotic translation initiation factor 5A-1 OS=Homo sapiens GN=EIF5A PE=1 SV=2 - | IF5A1 |
| Isoform 3 of Heterogeneous nuclear ribonucleoprotein K OS=Homo sapiens GN=HNRNPK - | HNRPK |
| Corneodesmosin OS=Homo sapiens GN=CDSN PE=1 SV=3 - | CDSN |
| Apolipoprotein D OS=Homo sapiens GN=APOD PE=1 SV=1 - | APOD |
| Far upstream element-binding protein 2 OS=Homo sapiens GN=KHSRP PE=1 SV=4 - | FUBP2 |
| 40S ribosomal protein S14 OS=Homo sapiens GN=RPS14 PE=1 SV=3 - | RS14 |
| Isoform 2 of Proteasome subunit alpha type-3 OS=Homo sapiens GN=PSMA3 - | PSA3 |
| Isoform Beta-1 of DNA topoisomerase 2-beta OS=Homo sapiens GN=TOP2B - | TOP2B |
| 60S ribosomal protein L4 OS=Homo sapiens GN=RPL4 PE=1 SV=5 - | RL4 |
| 40S ribosomal protein S6 OS=Homo sapiens GN=RPS6 PE=1 SV=1 - | RS6 |
| Proteasome subunit beta type-6 OS=Homo sapiens GN=PSMB6 PE=1 SV=4 - | PSB6 |
| Isoform 3 of Tetratricopeptide repeat protein 14 OS=Homo sapiens GN=TTC14 - | TTC14 |
| Isoform SV of 14-3-3 protein epsilon OS=Homo sapiens GN=YWHAE - | 1433E |
| Butyrophilin subfamily 1 member A1 OS=Homo sapiens GN=BTN1A1 PE=1 SV=3 - | BT1A1 |
| D-3-phosphoglycerate dehydrogenase OS=Homo sapiens GN=PHGDH PE=1 SV=4 - | SERA |
| Isoform 2 of Histone H2A.V OS=Homo sapiens GN=H2AFV - | H2AV |
| Histone H2AX OS=Homo sapiens GN=H2AFX PE=1 SV=2 - | H2AX |
| 60S ribosomal protein L35a OS=Homo sapiens GN=RPL35A PE=1 SV=2 - | RL35A |
| Fatty acid synthase OS=Homo sapiens GN=FASN PE=1 SV=3 - | FAS |
| Leucine-rich repeat-containing protein 59 OS=Homo sapiens GN=LRRC59 PE=1 SV=1 - | LRC59 |
| Isoform 3 of Protein odr-4 homolog OS=Homo sapiens GN=ODR4 - | ODR4 |
| 40S ribosomal protein S16 OS=Homo sapiens GN=RPS16 PE=1 SV=2 - | RS16 |
| Isoform 2 of F-actin-capping protein subunit alpha-2 OS=Homo sapiens GN=CAPZA2 - | CAZA2 |
| 60S ribosomal protein L5 OS=Homo sapiens GN=RPL5 PE=1 SV=3 - | RL5 |
| Proteasome subunit beta type-1 OS=Homo sapiens GN=PSMB1 PE=1 SV=2 - | PSB1 |
| Alpha-enolase OS=Homo sapiens GN=ENO1 PE=1 SV=2 - | ENOA |
| Serotransferrin OS=Homo sapiens GN=TF PE=1 SV=3 - | TRFE |
| Isoform 2 of Lysosome-associated membrane glycoprotein 1 OS=Homo sapiens GN=LAMP1 - | LAMP1 |
| Poly(rC)-binding protein 1 OS=Homo sapiens GN=PCBP1 PE=1 SV=2 - | PCBP1 |
| Isoform 2 of Proliferation-associated protein 2G4 OS=Homo sapiens GN=PA2G4 - | PA2G4 |
| Serpin H1 OS=Homo sapiens GN=SERPINH1 PE=1 SV=2 - | SERPH |
| Isoform B of Phosphate carrier protein, mitochondrial OS=Homo sapiens GN=SLC25A3 - | MPCP |
| Proteasome activator complex subunit 4 OS=Homo sapiens GN=PSME4 PE=1 SV=2 - | PSME4 |
| Isoform 3 of Chromatin target of PRMT1 protein OS=Homo sapiens GN=CHTOP - | CHTOP |
| Gem-associated protein 5 OS=Homo sapiens GN=GEMIN5 PE=1 SV=3 - | GEMI5 |
| Isoform 3 of mRNA-capping enzyme OS=Homo sapiens GN=RNGTT - | MCE1 |
| Eukaryotic initiation factor 4A-III OS=Homo sapiens GN=EIF4A3 PE=1 SV=4 - | IF4A3 |
| Polypyrimidine tract-binding protein 1 OS=Homo sapiens GN=PTBP1 PE=1 SV=1 - | PTBP1 |
| 60S ribosomal protein L23a OS=Homo sapiens GN=RPL23A PE=1 SV=1 - | RL23A |
| Protein lin-28 homolog A OS=Homo sapiens GN=LIN28A PE=1 SV=1 - | LN28A |
| Chloride intracellular channel protein 1 OS=Homo sapiens GN=CLIC1 PE=1 SV=4 - | CLIC1 |
| 60S ribosomal protein L34 OS=Homo sapiens GN=RPL34 PE=1 SV=3 - | RL34 |
| Dolichyl-diphosphooligosaccharide--protein glycosyltransferase subunit 1 OS=Homo sapiens GN=RPN1 PE=1 SV=1 - | RPN1 |
| Isoleucine--tRNA ligase, cytoplasmic OS=Homo sapiens GN=IARS PE=1 SV=2 - | SYIC |
| Protein BEX5 OS=Homo sapiens GN=BEX5 PE=1 SV=1 - | BEX5 |
| Isoform B of Endothelin B receptor OS=Homo sapiens GN=EDNRB - | EDNRB |
| Bloom syndrome protein OS=Homo sapiens GN=BLM PE=1 SV=1 - | BLM |
| Calpain-1 catalytic subunit OS=Homo sapiens GN=CAPN1 PE=1 SV=1 - | CAN1 |
| Probable ATP-dependent RNA helicase DDX46 OS=Homo sapiens GN=DDX46 PE=1 SV=2 - | DDX46 |
| DNA (cytosine-5)-methyltransferase 1 OS=Homo sapiens GN=DNMT1 PE=1 SV=2 - | DNMT1 |
| Isoform D of Constitutive coactivator of PPAR-gamma-like protein 1 OS=Homo sapiens GN=FAM120A - | F120A |
| Histone H3.1 OS=Homo sapiens GN=HIST1H3A PE=1 SV=2 - | H31 |
| Isoform 3 of Heterogeneous nuclear ribonucleoprotein H3 OS=Homo sapiens GN=HNRNPH3 - | HNRH3 |
| Isoform 2 of Heterogeneous nuclear ribonucleoprotein M OS=Homo sapiens GN=HNRNPM - | HNRPM |
| Isoform 4 of Heterogeneous nuclear ribonucleoprotein Q OS=Homo sapiens GN=SYNCRIP - | HNRPQ |
| Isoform Short of Heterogeneous nuclear ribonucleoprotein U OS=Homo sapiens GN=HNRNPU - | HNRPU |
| Isoform 2 of KH domain-containing, RNA-binding, signal transduction-associated protein 1 OS=Homo sapiens GN=KHDRBS1 - | KHDR1 |
| Lupus La protein OS=Homo sapiens GN=SSB PE=1 SV=2 - | LA |
| Isoform 3 of Nucleophosmin OS=Homo sapiens GN=NPM1 - | NPM |
| Ubiquitin thioesterase OTUB1 OS=Homo sapiens GN=OTUB1 PE=1 SV=2 - | OTUB1 |
| Isoform 2 of Polyadenylate-binding protein 1 OS=Homo sapiens GN=PABPC1 - | PABP1 |
| Isoform 2 of Polyadenylate-binding protein 4 OS=Homo sapiens GN=PABPC4 - | PABP4 |
| Isoform 6 of Poly(rC)-binding protein 2 OS=Homo sapiens GN=PCBP2 - | PCBP2 |
| Proteasome subunit alpha type-4 OS=Homo sapiens GN=PSMA4 PE=1 SV=1 - | PSA4 |
| Cellular retinoic acid-binding protein 2 OS=Homo sapiens GN=CRABP2 PE=1 SV=2 - | RABP2 |
| 60S ribosomal protein L10 OS=Homo sapiens GN=RPL10 PE=1 SV=4 - | RL10 |
| 60S ribosomal protein L26 OS=Homo sapiens GN=RPL26 PE=1 SV=1 - | RL26 |
| Adenosylhomocysteinase OS=Homo sapiens GN=AHCY PE=1 SV=4 - | SAHH |
| Splicing factor, proline- and glutamine-rich OS=Homo sapiens GN=SFPQ PE=1 SV=2 - | SFPQ |
| Serine/arginine-rich splicing factor 9 OS=Homo sapiens GN=SRSF9 PE=1 SV=1 - | SRSF9 |
| Steryl-sulfatase OS=Homo sapiens GN=STS PE=1 SV=2 - | STS |
| Transgelin-2 OS=Homo sapiens GN=TAGLN2 PE=1 SV=3 - | TAGL2 |
| Isoform 1 of Vinculin OS=Homo sapiens GN=VCL - | VINC |
| Isoform 2 of Annexin A4 OS=Homo sapiens GN=ANXA4 - | ANXA4 |
| Isoform 2 of Serine/arginine-rich splicing factor 3 OS=Homo sapiens GN=SRSF3 - | SRSF3 |
| Isoform 2 of 26S proteasome non-ATPase regulatory subunit 2 OS=Homo sapiens GN=PSMD2 - | PSMD2 |
| Isoform 3 of ATP-dependent RNA helicase DDX1 OS=Homo sapiens GN=DDX1 - | DDX1 |
| Actin-related protein 3C OS=Homo sapiens GN=ACTR3C PE=2 SV=1 - | ARP3C |
| ATP synthase subunit O, mitochondrial OS=Homo sapiens GN=ATP5O PE=1 SV=1 - | ATPO |
| Isoform 2 of Cullin-associated NEDD8-dissociated protein 1 OS=Homo sapiens GN=CAND1 - | CAND1 |
| Isoform 2 of F-actin-capping protein subunit beta OS=Homo sapiens GN=CAPZB - | CAPZB |
| Cyclin-dependent kinase 1 OS=Homo sapiens GN=CDK1 PE=1 SV=3 - | CDK1 |
| Claudin-6 OS=Homo sapiens GN=CLDN6 PE=1 SV=2 - | CLD6 |
| Isoform 3 of Chromosome transmission fidelity protein 18 homolog OS=Homo sapiens GN=CHTF18 - | CTF18 |
| Cytochrome c OS=Homo sapiens GN=CYCS PE=1 SV=2 - | CYC |
| Isoform 7 of Deleted in malignant brain tumors 1 protein OS=Homo sapiens GN=DMBT1 - | DMBT1 |
| Endoplasmic reticulum resident protein 29 OS=Homo sapiens GN=ERP29 PE=1 SV=4 - | ERP29 |
| Isoform 2 of 6-phosphofructo-2-kinase/fructose-2,6-bisphosphatase 2 OS=Homo sapiens GN=PFKFB2 - | F262 |
| Protocadherin Fat 2 OS=Homo sapiens GN=FAT2 PE=1 SV=2 - | FAT2 |
| Flap endonuclease 1 OS=Homo sapiens GN=FEN1 PE=1 SV=1 - | FEN1 |
| Stress-70 protein, mitochondrial OS=Homo sapiens GN=HSPA9 PE=1 SV=2 - | GRP75 |
| Histone H1x OS=Homo sapiens GN=H1FX PE=1 SV=1 - | H1X |
| 3-hydroxyacyl-CoA dehydrogenase type-2 OS=Homo sapiens GN=HSD17B10 PE=1 SV=3 - | HCD2 |
| Isoform 5 of Interleukin enhancer-binding factor 3 OS=Homo sapiens GN=ILF3 - | ILF3 |
| Importin-5 OS=Homo sapiens GN=IPO5 PE=1 SV=4 - | IPO5 |
| Macrophage migration inhibitory factor OS=Homo sapiens GN=MIF PE=1 SV=4 - | MIF |
| Nucleolar protein 56 OS=Homo sapiens GN=NOP56 PE=1 SV=4 - | NOP56 |
| Nuclear pore complex protein Nup93 OS=Homo sapiens GN=NUP93 PE=1 SV=2 - | NUP93 |
| Phosphoglycerate kinase 1 OS=Homo sapiens GN=PGK1 PE=1 SV=3 - | PGK1 |
| Membrane-associated progesterone receptor component 1 OS=Homo sapiens GN=PGRMC1 PE=1 SV=3 - | PGRC1 |
| Isoform 4 of Phosphatidylinositol 4-phosphate 5-kinase type-1 gamma OS=Homo sapiens GN=PIP5K1C - | PI51C |
| Peptidyl-prolyl cis-trans isomerase B OS=Homo sapiens GN=PPIB PE=1 SV=2 - | PPIB |
| Isoform 2 of Proteasome assembly chaperone 1 OS=Homo sapiens GN=PSMG1 - | PSMG1 |
| Isoform 5 of ELKS/Rab6-interacting/CAST family member 1 OS=Homo sapiens GN=ERC1 - | RB6I2 |
| 60S acidic ribosomal protein P2 OS=Homo sapiens GN=RPLP2 PE=1 SV=1 - | RLA2 |
| 39S ribosomal protein L15, mitochondrial OS=Homo sapiens GN=MRPL15 PE=1 SV=1 - | RM15 |
| Isoform 2 of Small nuclear ribonucleoprotein Sm D2 OS=Homo sapiens GN=SNRPD2 - | SMD2 |
| X-ray repair cross-complementing protein 5 OS=Homo sapiens GN=XRCC5 PE=1 SV=3 - | XRCC5 |
| Isoform 3 of High mobility group protein HMGI-C OS=Homo sapiens GN=HMGA2 - | HMGA2 |
| Isoform 2 of Replication factor C subunit 4 OS=Homo sapiens GN=RFC4 - | RFC4 |
| Aminoacyl tRNA synthase complex-interacting multifunctional protein 2 OS=Homo sapiens GN=AIMP2 PE=1 SV=2 - | AIMP2 |
| DNA-(apurinic or apyrimidinic site) lyase OS=Homo sapiens GN=APEX1 PE=1 SV=2 - | APEX1 |
| Isoform 3 of Apoptosis inhibitor 5 OS=Homo sapiens GN=API5 - | API5 |
| Chromatin assembly factor 1 subunit B OS=Homo sapiens GN=CHAF1B PE=1 SV=1 - | CAF1B |
| Isoform 1 of Histone-arginine methyltransferase CARM1 OS=Homo sapiens GN=CARM1 - | CARM1 |
| Probable ATP-dependent RNA helicase DDX6 OS=Homo sapiens GN=DDX6 PE=1 SV=2 - | DDX6 |
| Elongation factor 1-beta OS=Homo sapiens GN=EEF1B2 PE=1 SV=3 - | EF1B |
| Isoform 3 of Elongation factor 1-delta OS=Homo sapiens GN=EEF1D - | EF1D |
| Elongation factor Tu, mitochondrial OS=Homo sapiens GN=TUFM PE=1 SV=2 - | EFTU |
| Isoform EWS-B of RNA-binding protein EWS OS=Homo sapiens GN=EWSR1 - | EWS |
| Isoform 2 of DNA mismatch repair protein Msh2 OS=Homo sapiens GN=MSH2 - | MSH2 |
| Isoform 2 of DNA mismatch repair protein Msh2 OS=Homo sapiens GN=MSH2 - | MSH2 |
