# Supplementary material for: Alu retrotransposons modulate Nanog expression through dynamic changes in regional chromatin conformation via aryl hydrocarbon receptor
Source: Epigenetics Chromatin. 2020 Mar 14;13:15. doi: 10.1186/s13072-020-00336-w (PMC7071633; doi:10.1186/s13072-020-00336-w)
Supplement: Supplementary file 3 — Additional file 3: Table S3. Complete list of primers used in chIP, 3C, enchIP and CRISPR experiments. [file 13072_2020_336_MOESM3_ESM.docx]

**Supplementary table 2**

*Primers used for Alu elements ChIP experiments*

| Name | Sense | Sequence (5´-3´) |
| --- | --- | --- |
|  |  |  |
| ***NANOG x45s*** | *forward* | GCCAAAACCCTGTTTCCTTA |
|  | *reverse* | CGGCCTCTGCTCATCTTTTA |
|  |  |  |
| ***NANOG x45s-s*** | *forward* | AGGAGAATCGCTTGAACCTG |
|  | *reverse* | CGGCCTCTGCTCATCTTTTA |
|  |  |  |
| ***NANOG x14s*** | *forward* | CCACTGTTCCTGGCTCAAGT |
|  | *reverse* | TTCCAGACAGGAGCGAGACT |
|  |  |  |

*Primers used for mRNA RT-qPCR*

| Name | Sense | Sequence (5´-3´) |
| --- | --- | --- |
|  |  |  |
| ***GAPDH (h)*** | *forward* | GAAGGTGAAGGTCGGAGTC |
|  | *reverse* | GAAGATGGTGATGGGATTTC |
|  |  |  |
| ***SOX2*** | *forward* | TGCGAGCGCTGCACAT |
|  | *reverse* | TCATGAGCGTCTTGGTTTTCC |
|  |  |  |
| ***OCT4*** | *forward* | GGAGGAAGCTGACAACAATGAAA |
|  | *reverse* | GGCCTGCACGAGGGTTT |
|  |  |  |
| ***KLF4*** | *forward* | GTGTGGGTGGCTGTTCTTTT |
|  | *reverse* | TGACAGTCCCTGCTGTTCAG |
|  |  |  |
| ***LIF*** | *forward* | TCTCTTCATTTCCTATTACACAGCTC |
|  | *reverse* | AGAAGGCCTGGACCACCACACTTAT |
|  |  |  |
| ***NANOG*** | *forward* | GATTTGTGGGCCTGAAGAAA |
|  | *reverse* | AAGTGGGTTGTTTGCCTTTG |
|  |  |  |
| ***NOTCH1*** | *forward* | TGCCAGACCAACATCAAC |
|  | *reverse* | CTCATAGTCCTCGGATTGC |
|  |  |  |
| ***SHH*** | *forward* | AGGGTCGAGCAGTGGACATC |
|  | *reverse* | ATTTGGCCGCCACGGAGTT |
|  |  |  |
| ***WNT1*** | *forward* | ATCCATCTCTCCCACCTCCT |
|  | *reverse* | AGCAACCTCCTTTCCCACTT |

*Primers used for 3C experiments*

| Name | Sense | Sequence (5´-3´) |
| --- | --- | --- |
|  |  |  |
| ***3C-1*** | *forward* | GGCGAGCTTGTGGTCTTG |
|  | *reverse* | ------------------------------------- |
|  |  |  |
| ***3C-2*** | *forward* | CCATGAGATTAGATGACCTGGA |
|  | *reverse* | ------------------------------------- |
|  |  |  |
| ***3C-3*** | *forward* | AATTAAACTTATTTGCTCCTGGTG |
|  | *reverse* | ------------------------------------- |
|  |  |  |
| ***3C-4*** | *forward* | TGCTAGAGAATTTCAATTGGTTACCT |
|  | *reverse* | ------------------------------------- |
|  |  |  |
| ***3C-5*** | *forward* | TTGGTAAATATTGCATGCCTCCT |
|  | *reverse* | ------------------------------------- |
|  |  |  |
| ***3C-6*** | *forward* | GCACAGCTACAGTATGGGACAG |
|  | *reverse* | ------------------------------------- |
|  |  |  |
| ***3C-7*** | *forward* | GGCTGAGACAGGAGAATTGC |
|  | *reverse* | ------------------------------------- |
|  |  |  |
| ***3C-8*** | *forward* | TGGGGCAGCTCAATTTCTAT |
|  | *reverse* | ------------------------------------- |
|  |  |  |
| ***ENr313-1*** | *forward* | TCTTTACGGACAAGGCTGCT |
|  | *reverse* | TATTTCCAAAAGCCGGAGTG |
|  |  |  |
| ***ENr313-2*** | *forward* | TGAGGTCATGTCCTTTGCAG |
|  | *reverse* | CTCTCCCTCCTCCCATCTTC |
|  |  |  |
| ***GAPDH*** | *forward* | CGGCTACTAGCGGTTTTACG |
|  | *reverse* | AAGAAGATGCGGCTGACTGT |
|  |  |  |

*Primers used for me3H3K methylation marks ChIPs*

| Name | Sense | Sequence (5´-3´) |
| --- | --- | --- |
|  |  |  |
| ***NANOG K-1*** | *forward* | CTCCTGACCTCTTGGTCTGC |
|  | *reverse* | GGAGGCTAATGCACGAGAAC |
|  |  |  |
| ***NANOG K-2*** | *forward* | TTTGCTGGAGCCTTGATCTT |
|  | *reverse* | CCAGCCAGGATGCAGTTATT |
|  |  |  |
| ***NANOG K-3*** | *forward* | TCATTTTGGTGGGATTTGGT |
|  | *reverse* | GCTGGGTAAAATGAGGCTGA |
|  |  |  |
| ***NANOG K-4*** | *forward* | CCTGAAGGAGAGAAGCATGG |
|  | *reverse* | TCTGTCTGGCAGTTGTTTGC |
|  |  |  |
| ***NANOG K-5*** | *forward* | AGCCTGAGCGACAGAGAAAG |
|  | *reverse* | TCTGACTCAGCTTCCCGAAT |
|  |  |  |
| ***NANOG K-6*** | *forward* | CTTGACCTCACCATCCACCT |
|  | *reverse* | CCACAGCTCAGGTGACAGAA |
|  |  |  |

*Primers used for enChIP-3xFLAGdCas9 experiments*

| Name | Sense | Sequence (5´-3´) |
| --- | --- | --- |
|  |  |  |
| ***enChIP-1*** | *forward* | CAAGATCAGCCTGACCAACA |
|  | *reverse* | ATCTTGGCTCACTGCAACCT |
|  |  |  |
| ***enChIP-2*** | *forward* | GTCGGGAGTTCAAGATCAGC |
|  | *reverse* | AATCTTGGCTCACTGCAACC |
|  |  |  |
| ***enChIP-3*** | *forward* | AGGAGAATCGCTTGAACCTG |
|  | *reverse* | CGGCCTCTGCTCATCTTTTA |
|  |  |  |
| ***enChIP-4*** | *forward* | CAGGAGAATCGCTTGAACCT |
|  | *reverse* | CGGCCTCTGCTCATCTTTTA |
|  |  |  |
| ***enChIP-5*** | *forward* | CCAAAGGGCCAAGTAGTCAA |
|  | *reverse* | TCCCTAGGGCTACAGGTGTG |
